# Interface‐Driven Catalytic Enhancements in Nitrogen‐Doped Carbon Immobilized CoNi_2_S_4_@ReS_2_/CC Heterostructures for Optimized Hydrogen and Oxygen Evolution in Alkaline Seawater‐Splitting

**DOI:** 10.1002/advs.202413245

**Published:** 2024-12-24

**Authors:** Yanhui Lu, Zhengqiang Zhao, Xiaotong Liu, Xu Yu, Wenqiang Li, Chengang Pei, Ho Seok Park, Jung Kyu Kim, Huan Pang

**Affiliations:** ^1^ School of Chemistry and Chemical Engineering Yangzhou University Yangzhou Jiangsu 225002 P. R. China; ^2^ Key Laboratory of Function‐oriented Porous Materials College of Chemistry and Chemical Engineering Luoyang Normal University Luoyang 471934 P. R. China; ^3^ School of Chemical Engineering Sungkyunkwan University (SKKU) 2066 Seobu‐Ro Suwon 16419 Republic of Korea; ^4^ State Key Laboratory of Coordination Chemistry Nanjing University Nanjing 210023 P. R. China

**Keywords:** heterostructure, nitrogen‐doped carbon, rhenium disulfide, seawater splitting, strong interaction

## Abstract

The rational design of multicomponent heterostructure is an effective strategy to enhance the catalytic activity of electrocatalysts for water and seawater electrolysis in alkaline conditions. Herein, MOF‐derived nitrogen‐doped carbon/nickel‐cobalt sulfides coupled vertically aligned Rhenium disulfide (ReS_2_) on carbon cloth (NC‐CoNi_2_S_4_@ReS_2_/CC) are constructed via hydrothermal and activation approaches. Experimental and theoretical analysis demonstrates that the strong interactions between multiple interfaces promote electron redistribution and facilitate water dissociation, thereby optimizing *H adsorption energy for the hydrogen evolution reaction (HER). Meanwhile, the adsorption energies of oxygenated intermediates are balanced to reduce the thermodynamic barrier for the oxygen evolution reaction (OER). Consequently, NC‐CoNi_2_S_4_@ReS_2_/CC shows smaller overpotentials of 87 and 253 mV for HER and OER at 10 mA cm^−2^, with a lower Tafel slope and R_ct_ than control samples. Superior catalytic stability is confirmed by cyclic voltammetry (CV) for 1000 cycles and CA test for 56 h. Furthermore, NC‐CoNi_2_S_4_@ReS_2_/CC presents exceptional electrocatalytic activity in both alkaline water/seawater electrolytes. Stability assessments reveal that NC‐CoNi_2_S_4_@ReS_2_/CC maintains a highly catalytic activity in both water and seawater, owing to the corrosion‐resistant properties of the sulfur species at the interface. These findings highlight the importance of designing heterostructure electrocatalysts for clean hydrogen production.

## Graphical abstract

The growth of ReS_2_ nanosheets on MOF derived metal sulfides catalyst shows excellent catalytic activity for alkaline seawater splitting, attributing to the controllable morphological structure and the rational interfacial design. The heterostructure can inhibit the restacking of ReS_2_ nanosheets to expose abundant edge active sites, facilitate electron transfer and boost the active sites to improve the electrocatalytic performance.

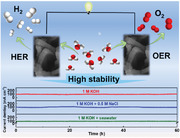

## 1 Introduction

Hydrogen energy is regarded as a viable alternative to fossil fuels due to its carbon‐neutral and zero‐emission properties, and electrochemical water splitting represents a promising and sustainable method for hydrogen production.^[^ ^1^ ^]^ However, efficient catalysts are essential to overcome the sluggish kinetics of oxygen and hydrogen formation and enhance the reaction rates at low overpotentials during the water dissociation process.^[^ ^2^ ^]^ Platinum group metals (PGMs)‐based composites, as ideal electrocatalysts for hydrogen evolution reaction (HER) and oxygen evolution reaction (OER), are restricted by their scarcity and high cost in large‐scale applications.^[^ ^3^ ^]^ Moreover, PGM‐based catalysts often suffer from stability issues under harsh operational conditions, including acidic or alkaline environments, which can lead to catalyst degradation and loss of activity.^[^ ^4^ ^]^ Additionally, their inherent properties may not be well‐suited for specific applications in seawater electrolysis. Therefore, it is urgent to explore the cost‐effective electrocatalyst with high catalytic stability as a promising alternative.

In recent years, a large number of nonnoble metal transition metal catalysts have emerged, mainly Fe‐, Co‐, and Ni‐based catalysts, including metal oxides,^[^ ^5^ ^]^ hydroxides,^[^ ^6^ ^]^ phosphides,^[^ ^7^ ^]^ sulfides,^[^ ^8^ ^]^ etc. They have been excavated to enhance the water‐splitting performance and reduce the catalyst cost, undoubtedly injecting vitality into the development of green hydrogen production. Transition metal sulfide (TMS) catalysts with good conductivity and high catalytic activity have attracted great attention.^[^ ^9^ ^]^ Therefore, TMS has been widely used in the field of electrocatalytic water splitting. Rhenium disulfide (ReS_2_) with a layered structure has attracted attention as an efficient catalyst due to its unique electronic properties and exposed active edge sites, and the restacking issue can be solved by growing ReS_2_ on the conductive supports.^[^ ^10^ ^]^ Compared with single‐component TMSs, multicomponent TMS nanocomposites with high‐density well‐designed heterogeneous interfaces have more active sites and exhibit better intrinsic catalytic activity.^[^ ^11^ ^]^ The electron transitions occur between metals with different valence states, which significantly improves the conductivity of the material.^[^ ^12^ ^]^ The CoS_2_‐ReS_2_ heterojunction electrocatalyst provides abundant edge catalytic sites and superior charge transfer performance during water cracking through the formed Re‐S‐Co interface engineering.^[^ ^10b^ ^]^ The introduction of metal ions to form polymetallic sulfide can adjust the electronic structure of the catalyst, and the synergy between polymetals is more conducive to electron transfer.^[^ ^13^ ^]^ Moreover, the design of a suitable spatial structure can reduce the aggregation of sulfides and expose more electron‐active sites.^[^ ^14^ ^]^ Core–shell has a larger specific surface area, which is more conducive to the exposure of active sites and provides more diffusion pathways for the transport of intermediates.^[^ ^15^ ^]^ The electron rearrangement was induced by the coupling interface between the Mo‐NiS shell and the NiTe nucleus, which promoted the transfer of electrons through the interface, and optimized the adsorption/decomposition of H_2_O, and the Mo‐NiS@NiTe heterojunction nanorod array had good reaction kinetics.^[^ ^16^ ^]^ The strong electronic interaction at the heterogeneous interface of the Co_3_S_4_@NiFe‐layered double hydroxides/NF core–shell structure promotes the transfer of electrons from NiFe‐LDH to Co_3_S_4_, reduces the energy barrier of the intermediates, and exhibits high electrocatalytic activity.^[^ ^17^ ^]^

Considering the sustainable production of hydrogen and the pressure on water resources, seawater electrolysis can be a long‐term goal for the construction of a hydrogen economy in the future.^[^ ^18^ ^]^ However, the presence of Cl^−^ in seawater makes this process still very challenging.^[^ ^19^ ^]^ The strong interaction between S atoms and metal atoms in TMS can resist the corrosion of Cl^−^ on metals, which is conducive to improving their corrosion resistance and chemical stability.^[^ ^20^ ^]^ NiFeS has excellent electron conductivity, and S‐species are rich in electrons, repel Cl^−^, enhance electrocatalytic performance, and exhibit excellent seawater spitting.^[^ ^21^ ^]^ The sulfate gradient layer generated by (Ni, Fe)OOH@NiCoS NAs is beneficial for improving the corrosion resistance and electrocatalytic performance of electrode materials by optimizing the coordination environment of chloride anions and hydroxyl intermediates.^[^ ^22^ ^]^ Moreover, the introduction of carbon materials derived from metal‐organic frameworks (MOF) can dramatically enhance the electrical conductivity and increase the porosity, which is favorable to improving the electrocatalytic activity of catalysts.^[^ ^17^ ^]^ Therefore, combining the above advantages is an effective way to prepare high‐efficiency water‐splitting electrocatalysts.

In this paper, a core–shell heterostructure composite catalyst (CoNi_2_S_4_@ReS_2_) with CoNiRe polymetallic sulfide/nitrogen‐doped carbon framework was successfully synthesized, with NC─CoNi_2_S_4_ as the core and rhenium disulfide (ReS_2_) as the shell, to achieve efficient electrocatalysis of overall water splitting. The N─C skeleton provided by MOF derivatization not only improves the active specific surface area of the material, but also improves the conductivity of the material, and the polymetallic CoNiRe excites more active sites in the metal sulfides. In the electrocatalytic test, the NC‐CoNi_2_S_4_@ReS_2_ heterostructure catalyst featured excellent HER and OER activities in alkaline fresh/seawater (1.0 m KOH) and maintained remarkable long‐term durability. In addition, the water‐splitting device, equipped with bifunctional NC‐CoNi_2_S_4_@ReS_2_/CC as both the cathode and anode, exhibited excellent performance with satisfactory stability. Additionally, density functional theory (DFT) calculations demonstrate that the heterogenous interface between NC─CoNi_2_S_4_ and ReS_2_ optimizes the hydrogen adsorption‐free energy and lowers the formation energy barrier of *OOH intermediates, which increases the HER and OER, respectively.

## 2 Results and Discussion

**Figure 1:**
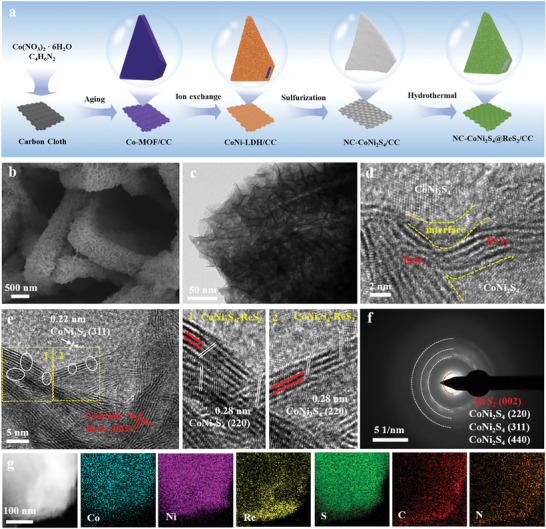
a) The synthesis process of heterostructured NC‐CoNi_2_S_4_@ReS_2_/CC composites. b) SEM, c) TEM, and d,e) high‐resolution TEM images, f) SAED pattern, and g) the corresponding elemental mapping of NC‐CoNi_2_S_4_@ReS_2_/CC.

The strategical synthesis of nitrogen‐doped carbon/cobalt‐nickel sulfides hybridized with ReS_2_ nanosheets on carbon cloth (NC‐CoNi_2_S_4_@ReS_2_/CC) is schematically illustrated in **Figure** **1**a. In brief, 2D cobalt‐based metal‐organic frameworks (2D Co‐MOF) with leaf‐shape morphology were directly grown on the surface of carbon cloth (CC), and subsequently converted to CoNi‐layered double hydroxides (Co‐LDH) via the ion exchange in Ni(NO_3_)_2_ aqueous solution. A hydrolysis‐controlled etching reaction is produced with the formation of hydrogen ions, which further etched the backbone of MOF and promoted the formation of CoNi‐LDH. After the thermal activation and hydrothermal treatment, CoNi_2_S_4_ grown on CC substrate was obtained by the ion exchange reaction with S^2−^. Finally, NC‐CoNi_2_S_4_@ReS_2_/CC heterostructure was constructed by vertically aligning ReS_2_ on NC‐CoNi_2_S_4_/CC, and ReS_2_ was formed by reducing ReO_4_^−^ with the help of S^2−^ and H^+^ during the hydrothermal process. The corresponding color change of all samples during the synthetic process is shown in Figure S1 (Supporting Information). The morphology of NC‐CoNi_2_S_4_@ReS_2_/CC was initially characterized by scanning electron microscopy (SEM). The carbon cloth substrate with smooth carbon fibers can provide a hierarchical structure for the growth of nanostructures, and the surface is decorated with 2D Co‐MOF nanosheets (Figure S2, Supporting Information). As treated by ion exchange, the 2D nanosheets are vertically aligned on the surface of Co‐MOF nanoleaf‐arrays (Figure S3, Supporting Information), and the roughness of the surface is increased due to the further sulfurized reaction with the well‐remained hierarchical structure (Figure S4, Supporting Information). Thin‐layer ReS_2_ nanosheets are vertically grown on the surface of CoNi_2_S_4_ nanoleaves under hydrothermal conditions (Figure 1b; Figure S5, Supporting Information), which guarantee the hierarchical structure of NC‐CoNi_2_S_4_@ReS_2_/CC. As verified by nitrogen adsorption/desorption isotherm in Figure S6 (Supporting Information), the specific surface area is 26.9 m^2^ g^−1^ with the average mesopore sites of 14.7 nm. In comparison, the direct growth of 2D ReS_2_ on CC for ReS_2_/CC shows a different morphology from NC‐CoNi_2_S_4_@ReS_2_/CC in Figure S7 (Supporting Information).

Transmission electron microscopy (TEM) was applied to evaluate the crystal structure of NC‐CoNi_2_S_4_@ReS_2_/CC in Figure 1c. It can be found that the thin‐layered ReS_2_ nanosheets are vertically grown without aggregation, which is consistent with the SEM result. High‐resolution TEM further reveals that the fringe spacings distances of 0.66, 0.28, and 0.22 nm correspond to the (002) plane of ReS_2_ and the (311) and (220) planes of CoNi_2_S_4_ (Figure 1d,e), respectively. Comparison to the pristine ReS_2_/CC (0.62 nm), the fringe spacing distance of ReS_2_ in NC‐CoNi_2_S_4_@ReS_2_ is increased (Figure S8, Supporting Information), suggesting that the interlayer spacing is expanded due to the formation of strong interaction between CoNi_2_S_4_ and ReS_2_. Notably, the interface between CoNi_2_S_4_ and ReS_2_, highlighted in the yellow squares and enlarged TEM images (marked with 1 and 2), exhibits excellent contact and well‐defined lattice fringes, which strongly supports the formation of NC‐CoNi_2_S_4_@ReS_2_ heterostructure. Selected area electron diffraction (SAED) shows clear diffraction rings corresponding to CoNi_2_S_4_ and ReS_2_ (Figure 1f), which is consistent with the HR‐TEM result. The strong interaction at the heterointerface can enhance the structural stability and promote the overall water‐splitting performance.^[^ ^23^ ^]^ Additionally, the TEM image and corresponding elemental mapping show the uniform distribution of Co, Ni, Re, S, C, and N for NC‐CoNi_2_S_4_@ReS_2_/CC (Figure 1g), which correlates with the energy‐dispersive X‐ray spectroscopy (EDS) results (Figure S9, Supporting Information), further confirming the successful fabrication of the NC‐CoNi_2_S_4_@ReS_2_ heterostructure.

**Figure 2:**
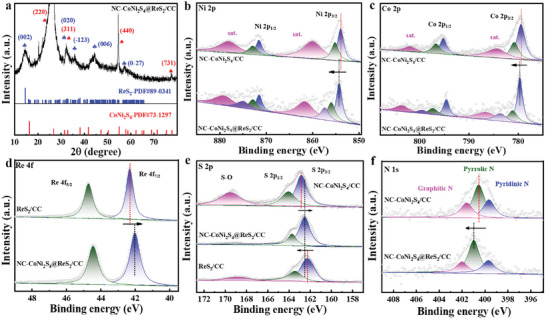
a) XRD result of NC‐CoNi_2_S_4_@ReS_2_/CC. b) Ni 2p and c) Co 2p spectra of NC‐CoNi_2_S_4_@ReS_2_/CC and NC‐CoNi_2_S_4_/CC. d) Re 4f spectra of NC‐CoNi_2_S_4_@ReS_2_/CC and ReS_2_/CC. e) S 2p spectra of NC‐CoNi_2_S_4_@ReS_2_/CC, NC‐CoNi_2_S_4_/CC, and ReS_2_/CC. f) N 1s spectra of NC‐CoNi_2_S_4_@ReS_2_/CC and NC‐CoNi_2_S_4_/CC.

X‐ray diffraction (XRD) was employed to analyze the crystal structure of NC‐CoNi_2_S_4_@ReS_2_/CC. As shown in **Figure** **2**a, the XRD pattern of NC‐CoNi_2_S_4_@ReS_2_/CC shows the apparent diffraction peaks at 14.5°, 32.3°, and 44.6° corresponding to (002), (020), and (006) planes the ReS_2_ (PDF#89‐0341), respectively. Additionally, the diffraction peaks at 26.7°, 32.2°, and 55.1° are attributed to the (220), (311), and (440) planes of CoNi_2_S_4_ (PDF#73‐1297). This observation confirms the successful formation of a heterojunction structure between CoNi_2_S_4_ and ReS_2_. The (002) peak of NC‐CoNi_2_S_4_@ReS_2_/CC shows a negative shift compared to pristine ReS_2_ (Figure S10, Supporting Information), attributing to the lattice expansion resulting from the formation of heterostructure at the ReS_2_/CoNi_2_S_4_ interface. Notably, the peak associated with the (220) and (440) plane of CoNi_2_S_4_ is particularly intense, which can be attributed to the growth of the catalyst on the carbon cloth (CC) substrate, aligning with the (002) and (100) plane of the carbon.^[^ ^24^ ^]^

The interfacial properties of heterojunction electrocatalysts are pivotal in modulating the electron‐state‐dependent catalytic activity, which directly influences overall performance. To probe the chemical composition and surface electronic state of NC‐CoNi_2_S_4_@ReS_2_/CC, X‐ray photoelectron spectroscopy (XPS) was employed. As the full XPS spectra are shown in Figure S11 (Supporting Information), NC‐CoNi_2_S_4_@ReS_2_/CC is composed of Co, Re, S, C, and N elements, and the small amount of N implies the successful heteroatom incorporation with the formation of C─N bond (Figure S12, Supporting Information). Binding energies were referenced to the C1s peak at 284.6 eV. Notably, the O1s spectrum exhibits no significant variation, suggesting minimal oxygen incorporation during synthesis (Figure S13, Supporting Information). N‐doped carbon, derived from Melm in ZIF‐derived LDH intermediates, enhances electron mobility across the carbon lattice and the sulfide nanostructure, promoting efficient charge transfer during the reaction.^[^ ^25^ ^]^ The high‐resolution Ni 2p spectrum reveals distinct peaks at 854.3/871.6 eV and 856.1/872.9 eV for NC‐CoNi_2_S_4_@ReS_2_/CC, corresponding to spin‐orbitals of Ni^2+^ and Ni^3+^ with a mixed valence state (Figure 2b). A negative shift of distinct peaks of Ni 2p can be found for NC‐CoNi_2_S_4_@ReS_2_/CC in contrast to the pristine NC‐CoNi_2_S_4_/CC catalyst. For Co 2p spectra in Figure 2c, the binding energies of 779.7, 794.6, 781.2, and 797.3 eV belong to the characteristic peaks of Co^3+^ and Co^2+^, respectively,^[^ ^26^ ^]^ which show similar negative shifts compared to NC‐CoNi_2_S_4_/CC. The mixed‐valence behavior of Ni and Co is crucial for optimizing redox dynamics at the catalytic interface. The formation of NC‐CoNi_2_S_4_@ReS_2_ heterointerface leads to a shift of Co 2p and Ni 2p peaks toward the high energy region, accompanied by the emergence of new chemical states of Ni/Co‐S‐Re between NC‐CoNi_2_S_4_ and layered ReS_2_ nanosheets. Figure 2d shows the Re 4f spectra and two primary peaks at 42.0 and 44.4 eV correspond to the Re 4f_7/2_ and Re 4f_5/2_, respectively.^[^ ^27^ ^]^ In comparison, Re 4f peaks shift to the low energy region, further confirming the formation of heterostructure. Peak shift in the Ni, Co, and Re spectra suggests the strong chemical coupling between the NC‐CoNi_2_S_4_ and ReS_2_ layers, and the interfacial charge transferring from CoNi_2_S_4_ to ReS_2_ due to the high electronegativity of the Re atom.^[^ ^10^, ^26^ ^]^ For S 2p spectra in Figure 2e, NC‐CoNi_2_S_4_@ReS_2_/CC shows two peaks at 162.5 and 163.7 eV corresponding to the spin‐orbital of S 2p_3/2_ and S 2p_1/2_, and the bond at 169.1 eV is ascribed to the oxidized sulfur species (─SO_x_). In comparison to NC─CoNi_2_S_4_/CC and ReS_2_/CC, an apparent peak shift of S 2p and the disappearance of ─SO_x_ bond for NC‐CoNi_2_S_4_@ReS_2_/CC results from the formation of heterointerfaces with the strong interaction at the interfaces, which is favorable to improving the catalytic performance. The divided N 1s spectra show three typical peaks of pyridine N, pyrrole N, and graphite N, which is derived from the conversion of Co─MOF to N‐doped carbon during the thermal activation treatment (Figure 2f). Pyridinic N has been reported to significantly enhance HER activity,^[^ ^28^ ^]^ and the pyrrolic N peak of NC‐CoNi_2_S_4_@ReS_2_/CC shifts toward higher energy following the incorporation of ReS_2_/CC. This result suggests that the introduction of the NC─CoNi_2_S_4_@ReS_2_ heterojunction alters the interaction between nitrogen and carbon atoms.^[^ ^29^ ^]^

**Figure 3:**
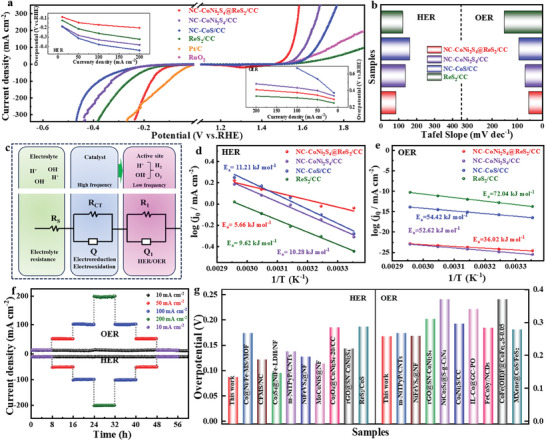
a) The polarization curves (insert: Overpotentials at 10, 50, 100, and 200 mA cm^−2^), b) Tafel slopes, c) electrical equivalent circuit model for analyzing the interfacial charge transfer, and d,e) Arrhenius plots (1/T vs log(j_0_) of NC‐CoNi_2_S_4_@ReS_2_/CC, NC‐CoNi_2_S_4_/CC, NC‐CoS/CC, and ReS_2_/CC. f) Long‐life durability of NC‐CoNi_2_S_4_@ReS_2_/CC. g) The comparison of overpotentials with reported HER/OER catalysts.

The electrocatalytic HER and OER performance of NC‐CoNi_2_S_4_@ReS_2_/CC in 1 m KOH was investigated by linear scanning voltammetry (LSV) in **Figure** **3**a. During the HER process, the overpotentials are 87, 188, 184, and 123 mV for NC‐CoNi_2_S_4_@ReS_2_/CC, NC‐CoS/CC, NC‐CoNi_2_S_4_/CC, and ReS_2_/CC at 10 mA cm^−2^, and the corresponding Tafel slopes are 83.7, 163.1, 136.8, and 122.9 mV dec^−1^ (Figure 3b), respectively. As the current density increased from 10 to 200 mA cm^−2^, NC‐CoNi_2_S_4_@ReS_2_/CC still owns the smallest overpotential. Even at a high current density, NC‐CoNi_2_S_4_@ReS_2_/CC shows a better catalytic HER performance than Pt/C. Meanwhile, a small overpotential of 253 mV at 10 mA cm^−2^ and a low Tafel slope of 54.7 mV dec^−1^ can be observed during the OER process. The smallest Tafel slope for NC‐CoNi_2_S_4_@ReS_2_/CC implies a fast electron transfer and catalytic reaction kinetics during the HER and OER processes.^[^ ^30^ ^]^ The presence of a heterogeneous interface, with its distinct physical and chemical properties, plays a critical role in enhancing catalytic performance. This interface forms strong interfacial bonds, creating an efficient pathway for electron transfer between different components. Furthermore, the heterostructure can modulate the adsorption energy of reaction intermediates, optimizing the interaction at the active sites and thereby boosting catalytic activity. Engineering surface chemistry not only accelerates reaction kinetics but also contributes to improved mass transfer efficiency.^[^ ^6a^ ^]^ To evaluate the intrinsic catalytic activity of all catalysts, the cyclic voltammetry (CV) curves in the non‐Faradaic region were measured at various scan rates for HER in Figure S14 (Supporting Information) and OER in Figure S15 (Supporting Information). The corresponding electrochemical double‐layer capacitance (C_dl_) was calculated (Figure S16, Supporting Information), and the electrochemical active surface area (ECSA) was integrated to study their specific catalytic activity (Tables S1 and S2, Supporting Information). By plotting the relationship between the current density and the scan rate for HER/OER, the C_dl_ values for NC‐CoNi_2_S_4_@ReS_2_/CC, NC‐CoNi_2_S_4_/CC, NC‐CoS/CC, and ReS_2_/CC are determined to be 54.0/42.9, 30.1/26.0, 16.0/17.2, and 34.2/12.0 mF cm^−2^, respectively. NC‐CoNi_2_S_4_@ReS_2_/CC owns the largest C_dl_ and ECSA values implying that the unique heterogeneous structure can expose an extended solid‐liquid interface and more active sites.

Turnover frequency (TOF) and the LSV normalized by ECSA were applied to explore the intrinsic electrochemical activity. As shown in Figure S17 (Supporting Information), both the TOF value and ECSA‐normalized current results demonstrate that NC‐CoNi_2_S_4_@ReS_2_/CC outperforms the control samples, resulting from the abundant exposure of active sits for facilitating efficient electron transfer and the formation of strong interfacial interactions for boosting the active sites. To monitor the adsorption kinetics and potential catalytic HER/OER mechanisms,^[^ ^31^ ^]^ electrochemical impedance spectroscopy (EIS) was applied, and the Nyquist plots in Figure S18 (Supporting Information) are fitted based on a hypothetical equivalent circuit model composed of three parts (Figure 3c). NC‐CoNi_2_S_4_@ReS_2_/CC with the low R_ct_ values elucidates the fast reaction kinetics (Tables S3 and S4, Supporting Information). The variation of electrocatalytic activity for NC‐CoNi_2_S_4_@ReS_2_/CC was further analyzed by investigating the effect of temperature on its catalytic performance (Figures S19 and S20, Supporting Information). Specifically, the apparent electrochemical activation energies (E_a_) for HER and OER were determined by the Arrhenius equation.^[^ ^32^ ^]^ As illustrated in Figure 3d,e, the slope of the Arrhenius plot (exchange current density, j_₀_ vs temperature, 1/T) demonstrates that NC‐CoNi_2_S_4_@ReS_2_/CC has the lowest energy barrier. Forming the heterojunction interface can effectively modulate the adsorption energy of transition state intermediates and significantly lower the kinetic energy barrier during the electrochemical process, thereby improving its overall electrocatalytic performance.^[^ ^32b^ ^]^ Long‐term durability, as a crucial parameter to evaluate the catalytic performance, was confirmed by CV curves for 1000 cycles and chronoamperometry (CA) test for 60 h. The negligible change of overpotential and current density probes exceptional catalytic stability of NC‐CoNi_2_S_4_@ReS_2_/CC during the HER/OER process in Figure 3f and Figure S21 (Supporting Information). Moreover, NC‐CoNi_2_S_4_@ReS_2_/CC possesses a superior catalytic activity to other reported transition metal sulfides‐based HER/OER catalysts (Figure 3g; Tables S5 and S6, Supporting Information).

The abovementioned results demonstrated that NC‐CoNi_2_S_4_@ReS_2_/CC shows excellent catalytic HER/OER performance, which is possibly attributed to the following reasons. 1) Hierarchical morphology can prevent the restacking of ReS_2_ nanosheets, provide more exposure to active sites and enhance the accessibility of reactant to the catalyst surface, resulting in improving reaction kinetics and promoting efficient mass transfer. 2) MOF‐derived nitrogen‐doped carbon enhances both the electronic conductivity and catalytic stability of the catalyst, while also serving as an active site for improved reaction performance. 3) Modulating surface chemistry by forming NC‐CoNi_2_S_4_@ReS_2_ heterostructure is beneficial to reduce the reaction energy barrier of water dissociation and adsorption/desorption of intermediates, thereby promoting the electrolysis of water.^[^ ^6^, ^33^ ^]^ 4) The strong interfacial electron interaction at the interface can facilitate more efficient charge transfer, thus boosting catalytic activity for both HER and OER during water splitting.

**Figure 4:**
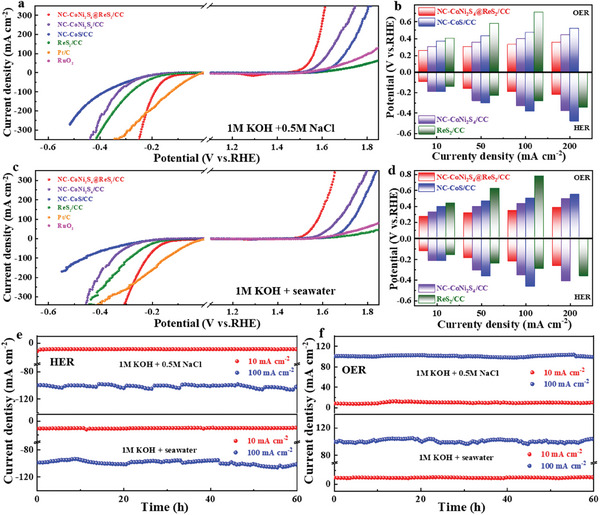
a,c) The polarization curves, b,d) overpotentials at 10, 50, 100, and 200 mA cm^−2^ of NC‐CoNi_2_S_4_@ReS_2_/CC, NC‐CoNi_2_S_4_ /CC, NC‐CoS/CC, and ReS_2_/CC. e,f) Long‐life durability of NC‐CoNi_2_S_4_@ReS_2_/CC in 1.0 m KOH+0.5 m NaCl and 1.0 m KOH+seawater.

Seawater electrolysis is attracting increasing attention due to the abundant availability of seawater, the cost‐effectiveness of hydrogen energy production, and the preservation of freshwater resources. However, seawater electrolysis faces significant challenges, including multiple competing reactions and a highly corrosive environment, which impose stringent requirements on catalyst performance. Considering the high electrocatalytic activity and durability of NC‐CoNi_2_S_4_@ReS_2_/CC in alkaline electrolytes, its electrochemical behavior in alkaline seawater electrolytes was further explored. **Figure** **4**a shows the LSV curves for HER and OER performance in simulated seawater (1 m KOH + 0.5 m NaCl), and NC‐CoNi_2_S_4_@ReS_2_/CC exhibits the lowest overpotential across the current density range from10 to 200 mA cm^−2^, indicating its superior catalytic activity (Figure 4b). Meanwhile, the values of Tafel slopes and C_dl_ for NC‐CoNi_2_S_4_@ReS_2_/CC are both smaller than that of control samples, elucidating a faster reaction dynamic during HER and OER process in the simulated seawater electrolyte (Figures S22–S25 and Tables S7 and S8, Supporting Information). Moreover, the overpotentials at different current densities, Tafel slopes, and C_dl_ values for NC‐CoNi_2_S_4_@ReS_2_/CC are much lower than those of control samples in real seawater (1 m KOH + seawater) electrolyte (Figure 4c,d; Figures S26–S29 and Tables S9 and S10, Supporting Information), implies that NC‐CoNi_2_S_4_@ReS_2_/CC facilitates faster charge transfer.^[^ ^34^ ^]^ Catalytic stability is a significant challenge for seawater‐splitting catalysts due to the corrosive ions present in seawater. To evaluate the long‐term durability of NC‐CoNi_2_S_4_@ReS_2_/CC, a CA test for 60 h was conducted at a fixed current in Figure 4e,f. NC‐CoNi_2_S_4_@ReS_2_/CC catalyst exhibits highly stable performance in both simulated alkaline and real seawater, maintaining a constant potential response for at least 50 h. This exceptional stability and corrosion resistance can be attributed to its robust support structure, which prevents the aggregation and loss of active sites in alkaline seawater solutions.^[^ ^1c^ ^]^ At the same time, the S‐species within the catalyst create a negatively charged, anion‐rich surface, effectively repelling Cl^−^ ions in seawater, thereby enhancing resistance to Cl^−^ induced corrosion.^[^ ^35^ ^]^ Additionally, the small diameters of semicircular observed in the Nyquist plot and the low R_ct_ values obtained from the fitted Nyquist plots imply that NC‐CoNi_2_S_4_@ReS_2_/CC facilitates fast charge transfer and rapid reaction kinetics during HER and OER process (Figures S30 and S31, Supporting Information).^[^ ^36^ ^]^ The activation energy reflects the kinetic energy barrier of catalyst during the OER process, and the NC‐CoNi_2_S_4_@ReS_2_/CC exhibits a low E_a_ value of 29.72 kJ mol^−1^ in alkaline seawater electrolyte (Figure S32, Supporting Information), indicating its favorable thermodynamic capability. These improvements are attributed to the optimization of adsorption energy and reaction kinetics, driven by interfacial electron interactions.

**Figure 5:**
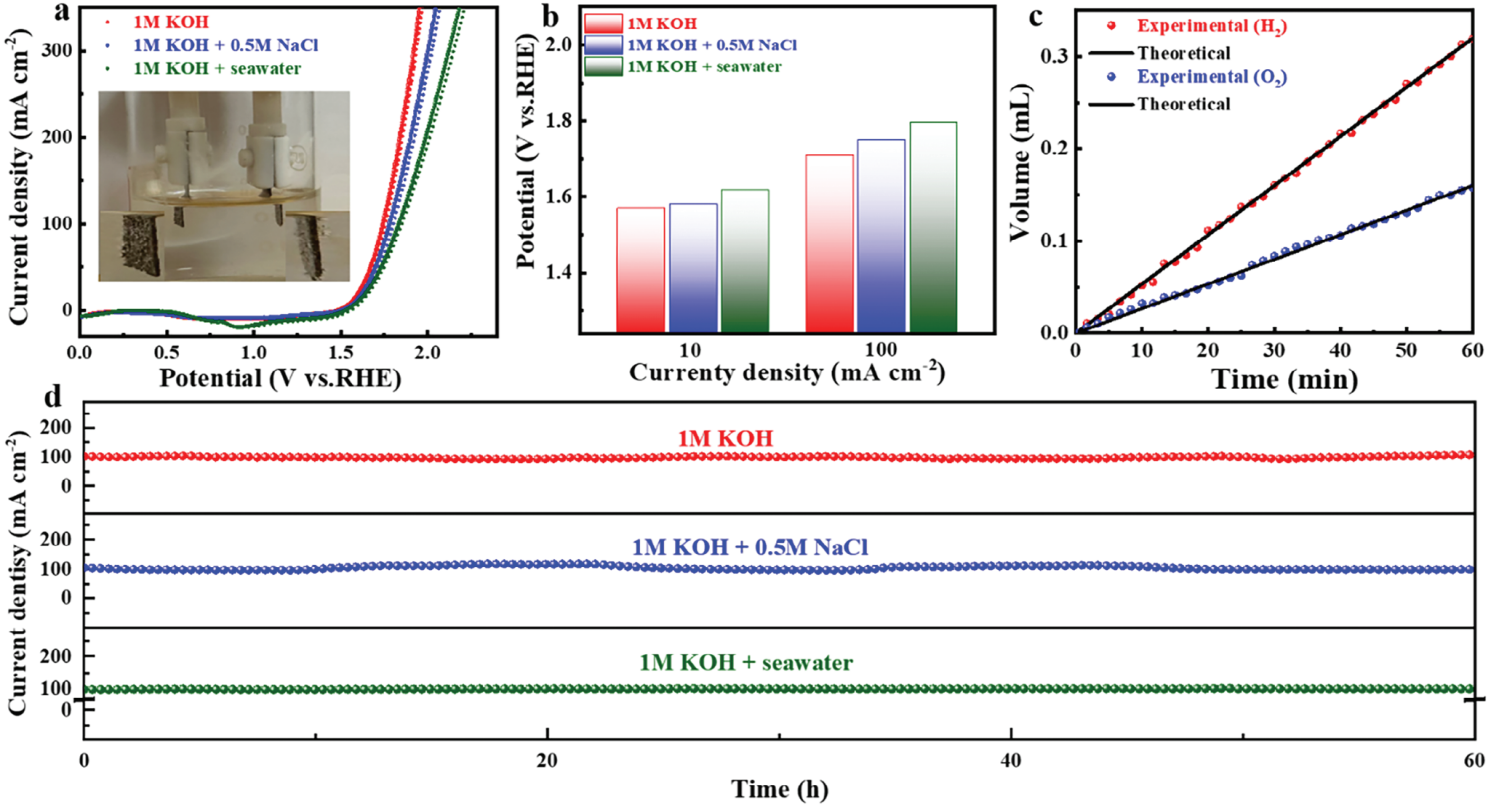
a) LSV curves of NC‐CoNi_2_S_4_@ReS_2_||NC‐CoNi_2_S_4_@ReS_2_ (insert: Optical image of electrolyzer for overall water splitting). b) Overpotentials at 10 and 100 mA cm^−2^ of NC‐CoNi_2_S_4_@ReS_2_/CC. c) Faradic efficiency of NC‐CoNi_2_S_4_@ReS_2_/CC in alkaline seawater. d) Chronoamperometry test of NC‐CoNi_2_S_4_@ReS_2_||NC‐CoNi_2_S_4_@ReS_2_ in different electrolytes.

The catalytic performance of NC‐CoNi_2_S_4_@ReS_2_/CC in pure alkaline, simulated seawater and real seawater electrolytes was investigated by assembling a symmetric NC‐CoNi_2_S_4_@ReS_2_/CC||NC‐CoNi_2_S_4_@ReS_2_/CC electrolyzer, and NC‐CoNi_2_S_4_@ReS_2_/CC was applied as both cathode and anode electrodes. **Figure** **5**a shows the polarization curves and the electrolyzer shows small cell voltages of 1.57 V at 10 mA cm^−2^ and 1.74 V at 100 mA cm^−2^ in pure alkaline electrolyte, which is superior to other reported catalysts in Table S11 (Supporting Information). It can be found that the hydrogen and oxygen bubbles are continuously produced at the respective electrodes during water splitting (inset of Figure 5a). Upon adding NaCl (1 m KOH + 0.5 m NaCl) to the electrolyte, the electrolyzer still maintains a high catalytic activity (Figure 5b), similar to its performance in pure 1 m KOH. However, the operating potential is increased to 1.83 V after introducing seawater to the pure alkaline electrolyte (1 m KOH + seawater). The enhanced potential is possibly attributed to the presence of impurities in seawater, such as chloride ions and other dissolved species, which can interfere with the electrochemical reaction, disrupt the catalytic process, and reduce the overall efficiency. Furthermore, the Faradaic efficiency of NC‐CoNi_2_S_4_@ReS_2_/CC in different electrolytes was assessed by comparing the theoretical and actual amounts of produced oxygen/hydrogen over 60 min at a specific overpotential (Figure 5c; Figure S33, Supporting Information). The production of hydrogen and oxygen is closely aligned with theoretical values, indicating a Faradaic efficiency of ≈100% for both HER and OER. Additionally, no apparent degradation of current density can be found for the NC‐CoNi_2_S_4_@ReS_2_/CC electrolyzer after the CA test for 60 h, highlighting its exceptional stability throughout the water‐splitting process (Figure 5d). The bifunctional nature of NC‐CoNi_2_S_4_@ReS_2_/CC with excellent catalytic performance positions it as a promising candidate for the cost‐effective and energy‐efficient catalyst for water splitting.

**Figure 6:**
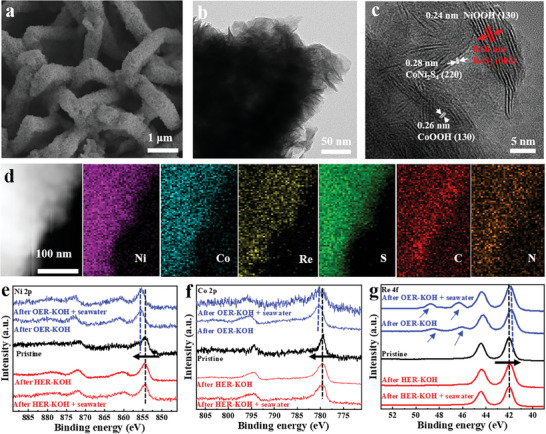
a) SEM, b)TEM, and c) HR‐TEM images and d) elemental mapping of NC‐CoNi_2_S_4_@ReS_2_/CC catalyst after CA test in alkaline seawater solution. High‐resolution e) Ni 2p, f) Co 2p, and g) Re 4f spectra of NC‐CoNi_2_S_4_@ReS_2_/CC catalyst after HER and OER stability test in different electrolytes.

The change of morphological and crystal structure for NC‐CoNi_2_S_4_@ReS_2_/CC was characterized by SEM and TEM after catalytic stability tests in alkaline seawater electrolytes. As shown in **Figure** **6**a, NC‐CoNi_2_S_4_@ReS_2_/CC largely retains the original morphology and the increased surface roughness can be ascribed to the surface corrosion and the formation of metal oxides. As can be seen from HR‐TEM images (Figure 6b), the surface of the catalyst becomes dark. The lattice spacings of 0.24, 0.28, 0.26, and 0.66 nm correspond to (130) planes of NiOOH (PDF#06‐0075), (220) plane of CoNi_2_S_4_ (PDF#73‐1297), (130) plane of CoOOH (PDF#26‐0480), and (002) plane of ReS_2_ (PDF#89‐0341) in Figure 6c, respectively. Figure 6d shows the elemental mapping images of NC‐CoNi_2_S_4_@ReS_2_/CC and the distribution of all elementals is uniform. These results confirm that ReS_2_ nanosheets maintain a high degree of crystallinity and the surface of CoNi_2_S_4_ undergoes partial reconstruction to form corresponding hydroxides and oxides.^[^ ^26a^ ^]^ As confirmed by the EDS result in Figure S34 (Supporting Information), the additional elements (Na, Cl, K, etc) are from the adsorption of residual electrolytes on the surface of catalyst. The change of surface chemical environment for NC‐CoNi_2_S_4_@ReS_2_/CC after the electrochemical stability test was probed by XPS in Figure 6e–g. The full XPS spectra further confirm the presence of few impurities on the surface of catalyst (Figure S35, Supporting Information), which is well‐matched with the EDX result. In comparison to the pristine state of catalyst, no apparent peak shift of high resolution of Ni 2p, Co 2p, and Re 4f for NC‐CoNi_2_S_4_@ReS_2_/CC can be observed after HER test in alkaline freshwater/seawater electrolytes, and a slight positive shift of Co and Ni spectra after OER test can be attributed to the surface modulation with forming oxygen‐containing intermediates, which is consistent with the HRTEM result. In particular, the Re peak after the OER test is slightly shifted to a lower binding energy, and the appearance of additional two peaks correspond to the valence transition from Re─S to Re─O bonds, which is further confirmed by S spectra in Figure S36 (Supporting Information). Negligible oxidation was observed after the HER test, while the increase in sulfur─oxygen bonds following the OER test is attributed to surface reconstruction during the long‐term stability test. This process facilitates the formation of active oxide or hydroxide phases, in agreement with the HR‐TEM results discussed above.^[^ ^10^, ^27^, ^37^ ^]^

**Figure 7:**
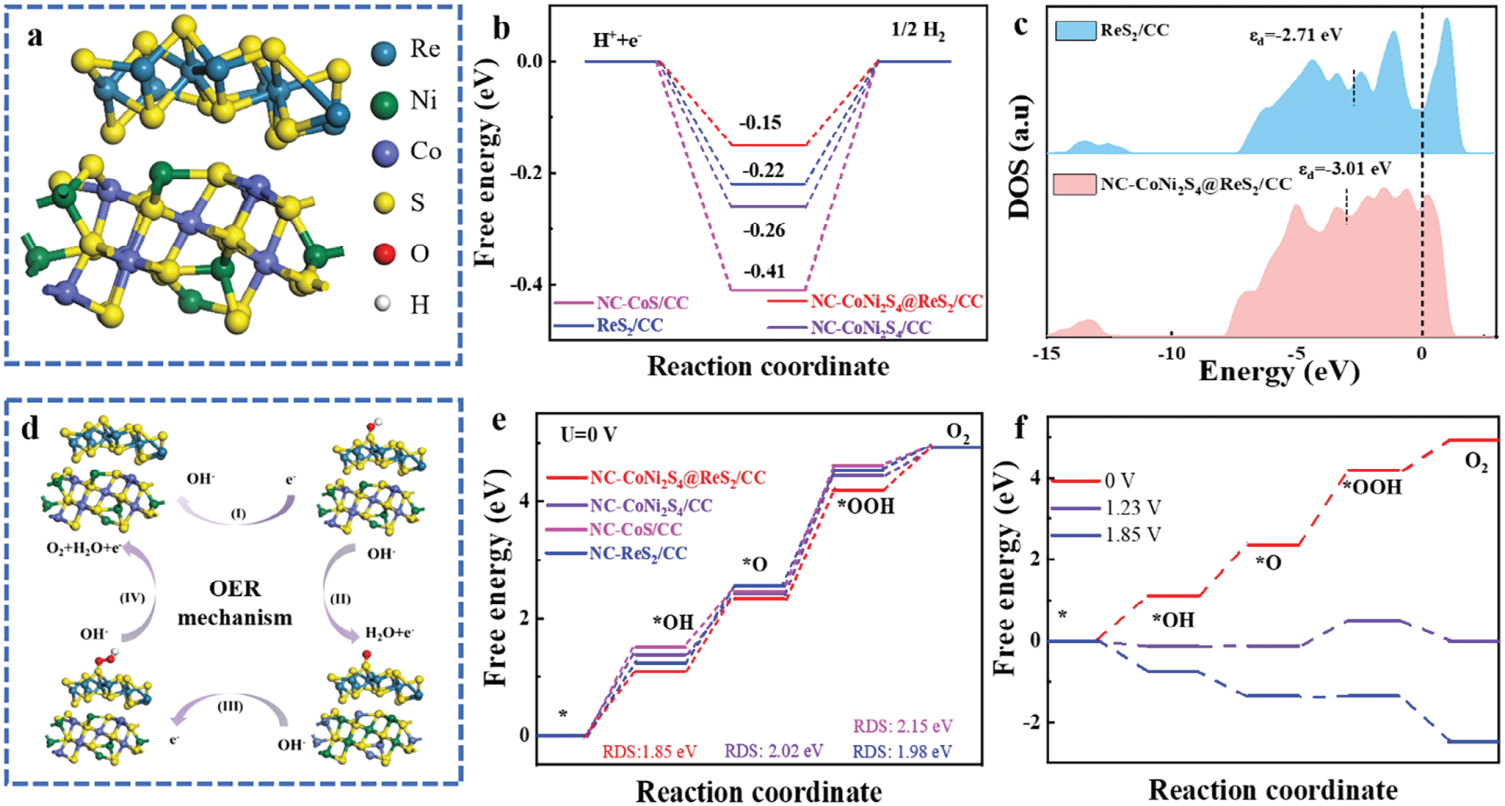
a) The simulated calculation models. b) Gibbs free energy diagrams for HER of different electrocatalysts. c) The calculated d‐band center of NC‐CoNi_2_S_4_@ReS_2_/CC and ReS_2_/CC. d) Schematic mechanisms obtained by DFT analysis for OER in terms of adsorption of *OH, *O, and *OOH on the NC‐CoNi_2_S_4_@ReS_2_/CC. e) Adsorption energy of oxygen intermediates on different electrocatalysts. f) The calculated free energy diagram of OER at U = 0, 1.23, and 1.85 V.

To elucidate the intrinsic contribution and catalytic mechanism of NC‐CoNi_2_S_4_@ReS_2_ heterointerface, density functional theory (DFT) computation was carried out. According to the HRTEM results, the (311) plane of CoNi_2_S_4_ and (002) plane of ReS_2_ as the dominant active site were selected to simulate the models of catalysts (**Figure** **7**a; Figure S37, Supporting Information). It is well known that hydrolytic ionization and hydrogen adsorption are the primary steps during the HER process in alkaline electrolytes, and the activation energy barrier of water decomposition is a critical factor in influencing the HER kinetics. Gibbs free energy of hydrogen adsorption (ΔG_*H_) is an important parameter for evaluating the HER activity, and the value is close to 0 (ΔG_*H_ ≈ 0) indicating an optimal balance between hydrogen adsorption and desorption.^[^ ^38^ ^]^ The optimal adsorption sites and ΔG_*H_ diagram for intermediates were determined through calculations, and the optimized structure of hydrogen adsorption on NC‐CoNi_2_S_4_@ReS_2_ heterointerface is presented in Figure 7b and Figure S38 (Supporting Information). NC‐CoNi_2_S_4_@ReS_2_/CC exhibits the lowest ΔG_*H_ at −0.15 eV, which is close to the ideal value of 0 eV, indicating more favorable hydrogen adsorption kinetics and superior HER activity. Additionally, the d‐band center (ɛ_d_) of Re based on the project density of state (PDOS) analysis is illustrated in Figure 7c, and the PDOS of the NC‐CoNi_2_S_4_@ReS_2_/CC exhibits a significantly higher occupancy near the Fermi energy (E_f_) compared to ReS_2_, suggesting a strong interaction at the heterointerface between NC‐CoNi_2_S_4_ and ReS_2_. The hybridization changes the d‐electron configuration near E_f_, resulting in faster electron transfer and higher conductivity, which enhances the catalytic performance of NC‐CoNi_2_S_4_@ReS_2_/CC in the HER process.^[^ ^39^ ^]^ For NC‐CoNi_2_S_4_@ReS_2_/CC, the *ɛ*_d_ value of Re (−3.01 eV) is shifted to a lower energy level compared to ReS_2_ (−2.71 eV). According to d‐band center theory, a lower d‐band energy level relative to E_f_ leads to more filled antibonding states, resulting in weaker adsorption bonds. The downward shift of the d‐band center weakens hydrogen adsorption on the catalyst surface, thereby reducing the activation barrier for HER and improving the electrocatalytic activity.^[^ ^40^ ^]^

The OER process includes four‐electron reaction mechanisms, and the ΔG of oxygen‐containing intermediates (*OH, *O, *OOH) is used to estimate electrocatalytic activity. The evaluation of catalytic OER reaction mainly depends on the rate determination step (RDS), which reflects the maximum energy barrier required by intermediates reaction.^[^ ^41^ ^]^ Figures S39–S42 (Supporting Information) show the adsorption of OER intermediates on the simulated crystal surface of all the catalysts from the. For the catalytic OER process in alkaline electrolytes, the conversion of intermediates adsorption with the electron transfer is illustrated in Figure 7d. The Gibbs free energy for each fundamental step is favorable to elucidate the catalytic activity, and the formation of *OOH intermediates with the largest Gibbs free energy is identified as the RSD during the OER process. For the conversion of intermediates from *O to *OOH (Figure 7e), the ΔG value of NC‐CoNi_2_S_4_@ReS_2_/CC (1.85 eV) is smaller than that of NC‐CoNi_2_S_4_/CC (2.02 eV), NC‐CoS/CC (2.15 eV), and ReS_2_/CC (1.98 eV), suggesting that NC‐CoNi_2_S_4_@ReS_2_ heterointerface facilitates charge transfer between NC‐CoNi_2_S_4_ and ReS₂, thereby enhancing the interaction between the catalyst and OER intermediates. At the potential energy U is 0 V, NC‐CoNi_2_S_4_@ReS_2_/CC shows the thermodynamical surface for all four steps in Figure 7f. However, a noticeable energy barrier still exists from step 3 to step 4 during the OER process at U of 1.23 V. As U increases to 1.85 V, a theoretical overpotential is ≈0.62 V and the maximum ΔG for the fundamental OER step is close to 0 eV, indicating that the entire OER process can proceed spontaneously at this potential.^[^ ^36^, ^42^ ^]^ These theoretical results demonstrate that the NC‐CoNi_2_S_4_@ReS_2_ heterointerface effectively optimizes the electronic structure of the catalyst, reducing the thermodynamic barriers for both hydrogen adsorption in HER and *OOH formation in OER, thus enhancing overall catalytic performance.

## 3 Conclusion

In this study, we developed a hierarchical NC‐CoNi_2_S_4_@ReS_2_ heterointerface with a core–shell structure, demonstrating its high efficiency as a bifunctional electrocatalyst for both HER and OER in water/seawater splitting. The synergistic interaction at the NC‐CoNi_2_S_4_@ReS₂ heterointerface significantly enhances the catalytic performance by optimizing the electronic structure, thereby facilitating faster charge transfer and improving conductivity. The core–shell architecture not only provides a large surface area and abundant active sites but also ensures the stability of the catalyst by preventing aggregation and deactivation of active sites under harsh conditions. NC‐CoNi_2_S_4_@ReS_2_/CC exhibits excellent HER and OER activities, with a low overpotential, high current density, and near‐ideal Gibbs free energy for hydrogen adsorption (ΔG_*H_). Moreover, the NC‐CoNi_2_S_4_@ReS_2_ interface showed enhanced stability and corrosion resistance in both alkaline and seawater environments, resulting from repelling corrosive Cl^−^ ions in seawater by the negatively charged sulfur species. The OER The conversion from *O to *OOH intermediates shows a lower energy barrier during the OER process, contributing to the superior oxygen evolution performance.

## Conflict of Interest

The authors declare no conflict of interest.

## Supporting information



Supporting Information

## Data Availability

The data that support the findings of this study are available from the corresponding author upon reasonable request.
