# Supplementary material for: Interface‐Driven Catalytic Enhancements in Nitrogen‐Doped Carbon Immobilized CoNi2S4@ReS2/CC Heterostructures for Optimized Hydrogen and Oxygen Evolution in Alkaline Seawater‐Splitting
Source: Adv Sci (Weinh). 2024 Dec 24;12(7):2413245. doi: 10.1002/advs.202413245 (PMC11831512; doi:10.1002/advs.202413245)
Supplement: Supplementary file 1 — Supporting Information [file ADVS-12-2413245-s001.docx]

**Supporting Information**

**Interface-driven Catalytic Enhancements in Nitrogen Doped Carbon Immobilized CoNi_2_S_4_@ReS_2_/CC Heterostructures for Optimized Hydrogen and Oxygen Evolution in Alkaline Seawater-splitting**

Yanhui Lu ^a^, Zhengqiang Zhao ^a^, Xiaotong Liu ^a^, Xu Yu ^a,^ *, Wenqiang Li ^b,^ *, Chengang Pei ^c^, Ho Seok Park ^c^, Jung Kyu Kim ^c^, and Huan Pang ^a,^ *

Y. Lu, Z. Zhao, X. Liu, X. Yu, H. Pang

School of Chemistry and Chemical Engineering, Yangzhou University, Yangzhou, Jiangsu 225002, P. R. China

E-mail: yxypz15@yzu.edu.cn, panghuan@yzu.edu.cn

W. Li

Key Laboratory of Function-oriented Porous Materials, College of Chemistry and Chemical Engineering, Luoyang Normal University, Luoyang 471934, P. R. China

E-mail: liwenqiang@lynu.edu.cn

C. Pei, H. S. Park, J. K. Kim

School of Chemical Engineering, Sungkyunkwan University (SKKU), 2066 Seobu-Ro, Suwon 16419, Republic of Korea

**S1. Experimental Section**

S1.1 Materials:

Carbon cloth, Nickel nitrate hexahydrate [Ni(NO_3_)_2_·6H_2_O, AR], Cobalt nitrate hexahydrate [Co(NO_3_)_2_·6H_2_O, AR], 2-Methylimidazole, hydroxylamine hydrochloride (HONH_2_HCl), thiourea (CH_4_N_2_S) and sodium sulfide nonahydrate (Na_2_S·9H_2_O) were purchased from Shanghai Aladdin Biochemical Technology Co., Ltd. Ammonium perrhenate (NH_4_ReO_4_) was purchased from Guangdong Wengjiang Chemical Reagent Co., Ltd. Nafion (5 wt%) and ethanol was purchased from Sigma-Aldrich Co. All chemicals were used without further purification.

S1.2.1 Preparation of NC-CoNi_2_S_4_/CC

Cobalt nitrate hexahydrate (0.1 mmol) and 2-Methylimidazole (8 mmol) were dissolved in 40 mL of deionized (DI) water under magnetic stirring for 10 min, and then a piece of treated carbon cloth (1 cm*4 cm) was immersed in the solution and kept for 6 h. The sample was carefully washed with DI water, heated at 60 °C overnight, and named as Co-MOF/CC. After that, the as-prepared Co-MOF/CC was immersed in 40 mL of Ni(NO_3_)_2_·6H_2_O solution (0.1 mmol) for 6 h, washed with DI water several times, and named as CoNi-LDH/CC. Subsequently, the CoNi-LDH/CC sample was heated at 350 ^o^C for 2 h with a heating rate of 2 ^o^C min^-1^ under air atmosphere. Finally, the sample was immersed in 30 mL of Na_2_S solution (0.1 mol) and transferred to a 50 mL of stainless steel autoclave via hydrothermal reaction at 90 °C for 9 h, named NC- CoNi_2_S_4_/CC.

S1.2.2 Preparation of NC-CoNi_2_S_4_@ReS_2_/CC

0.1 mmol of ammonium perrhenate, 1 mmol of thiourea, and 0.6 mmol of hydroxylamine hydrochloride were mixed in 30 mL of DI water to make a homogeneous solution and transferred to a 50 mL of stainless steel autoclave. Subsequently, the as-prepared NC-CoNi_2_S_4_/CC was immersed in the solution and hydrothermally reacted at 220 °C for 10 h. The sample was finally washed with DI water, dried at 60 ^o^C overnight in a vacuum oven, and named NC-CoNi_2_S_4_@ReS_2_/CC.

S1.2.3 Preparation of NC-CoS/CC:

Co-MOF/CC sample was heated at 350 ^o^C for 2 h with a heating rate of 2 ^o^C min^-1^ under air atmosphere. Finally, the sample was immersed in to 30 mL of Na_2_S solution (0.1 mol) and transferred to a 50 mL of stainless steeled autoclave via hydrothermal reaction at 90 °C for 9 h, named NC-CoS/CC.

S1.2.4 Preparation of ReS_2_/CC:

0.1 mmol of ammonium perrhenate, 1 mmol of thiourea, 0.6 mmol of hydroxylamine hydrochloride were mixed in 30 mL of DI water to make a homogeneous solution and transferred to a 50 mL of stainless steeled autoclave. Subsequently, the as-prepared carbon cloth was immersed in the solution and hydrothermally reacted at 220 °C for 10 h. The sample was finally washed with DI water, dried at 60 ^o^C overnight in a vacuum oven, and named ReS_2_/CC.

**S2. Characterization**

Powder X-ray diffraction (XRD) patterns were recorded on a Bruker D8 Advance powder X-ray diffractometer using a Cu Kα (λ = 1.5405 Å) radiation source operating at 40 kV and 40 mA with the scanning rate of 5° min^-1^. The morphology and microstructure of the product were analyzed by scanning electron microscopy (FESEM, Hitachi, S-4800 II, Japan) and transmission electron microscopy (TEM, Philips, TECNAI 12, Holland). The specific surface area was measured through an ASAP 2460 system (Micromeritics) using the Brunauer-Emmett-Teller (BET) method. All X-ray photoelectron spectroscopy (XPS) measurements were carried out on Kratos XSAM-800 spectrometers with an Al Kα radiation source.

**S3. Electrochemical measurements**

The electrochemical measurements were performed in a 1.0 M KOH electrolyte at room temperature using an electrochemical workstation (CHI660E) by a three-electrode system. The as-prepared NC-CoNi_2_S_4_@ReS_2_/CC (1.65 mg cm^-2^) is the working electrode. Graphite rod and saturated calomel electrode (SCE) were used as the counter and reference electrodes, respectively. All potentials were referenced to a reversible hydrogen electrode (RHE), the formula is as follows: E(RHE) = E(SCE) + 0.0591*pH + 0.242V.

The polarization curves were measured by cyclic voltammetry (CV) at a scan rate of 5 mV s^-1^. The ohmic resistance with IR (90%) correction was obtained using electrochemical impedance spectroscopy in the frequency range of 1000 kHz~10 mHz with an amplitude of 5 mV. The durability test was carried out for 1000 cyclic voltammetry (CV) cycles at a scan rate of 150 mV s^-1^, and a linear sweep was measured at the scan rate of 5 mV s^-1^ after 1000 cycles. The cyclic voltammetry (CV) curves were conducted to calculate the electrochemical active surface areas (ECSA) and tested under various scan rates at different scan rates in a non-Faradaic region, which is proportional to the double layer capacitance (C_dl_). The ECSA can be estimated according to the equation:

$$ECSA=\frac{C_{dl}}{C_{s}}$$

LSV normalized by ECSA was carried out to explore the intrinsic activity of active sites for each sample based on the equation:

$$j_{ECSA-normalized}=j*\frac{C_{s}}{C_{dl}}$$

Where the specific capacitance (C_s_) is the specific capacitance at 1 M KOH, and the widely used 0.04 mF·cm^-2^ was used here.

A chronoamperometry (CA) test was carried out at the overpotential for HER and OER in both alkaline fresh/seawater media. After the CA test, the TEM and XPS of the active catalyst were carried out.

The Turnover frequency (TOF) values were calculated from the equation (assuming all metal ions as active sites):

$$TOF=j*\frac{A}{2(4)*F*m}$$

Where j is the current density at a specific potential, A is the surface area of the electrode, F is the Faraday constant (96485 C mol^-1^) and m is the number of moles of active materials. The Faradaic efficiency of NC-CoNi_2_S_4_@ReS_2_/CC was measured at different overpotentials for 1 h and calculated by the following equation.

$$Faradaic yield=\frac{V_{Exp}}{V_{Theor}}=\frac{V_{Exp}}{\frac{1(2)}{4}*\frac{Q}{F}*V_{m}}$$

Where the V_Exp_ and V_Theor_ are the experimental and theoretic volumes of the generated O_2_ gas during the catalytic process, Q is the charge passed through the electrode, F is Faraday constant (96485 C mol^-1^), the number 4 means 4-mole electrons per mole O_2_ (the number 1 (2) represents 1 mol of O_2_ (2 mol H_2_)), V_m_ is the molar volume of gas (24.5 L mol^-1^, 298 K, 101 KPa).

The apparent electrochemical activation energy (E_a_) for HER/OER can be determined using the Arrhenius plots, according to the equation:

$$\frac{\partial log(j_{0})}{\partial(1/T)}=-\frac{E_{a}}{2.303R}$$

Where j_0_ is the exchange current density, R is the universal gas constant (8.314 J mol ^-1^ K^-1^) and T is the absolute temperature.

**S4. Computational Methodology**

Spin-polarized density functional theory (DFT) calculations were carried out using the Vienna ab initio simulation package (VASP). The plane-wave basis set with an energy cutoff of 400 eV, the projector-augmented wave (PAW) potentials and the generalized gradient approximation (GGA) exchange-correlation functional parameterized by Perdew-Burke-Ernzerhof (PBE) were employed [1]. Take valence electrons into account using a plane wave basis set with a kinetic energy cutoff of 450 eV. Partial occupancies of the Kohn−Sham orbitals were allowed using the Gaussian smearing method and a width of 0.05 eV. The electronic energy was considered self-consistent when the energy change was smaller than 10^-5^ eV. A geometry optimization was considered convergent when the energy change was smaller than 0.05 eV Å^-1^. The Brillouin zone is sampled with 3 × 3 × 1 Monkhorst mesh.

The reaction Gibbs free energy changes (∆G) for each elementary step for HER and OER processes obtained by frequency calculations as described below:

$$\Delta G=\Delta E+{\Delta E}_{ZPE}-T\Delta S$$

where ΔE is obtained directly from DFT calculations, ΔE_ZPE_ is the change of zero-point energies, T is the temperature of 298.15 K, and ΔS is the change in entropy of products and reactants.


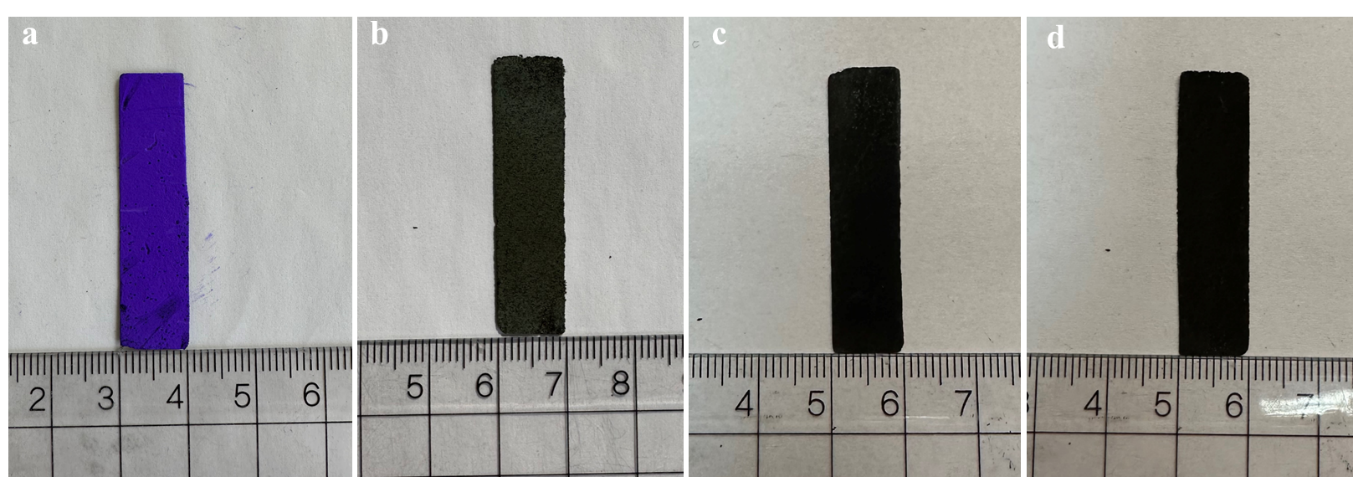


**Figure S1.** Prepared catalysts of the (a) Co-MOF/CC, (b) CoNi-LDH/CC, (c) NC-CoNi_2_S_4_/CC, (d) NC-CoNi_2_S_4_@ReS_2_/CC.


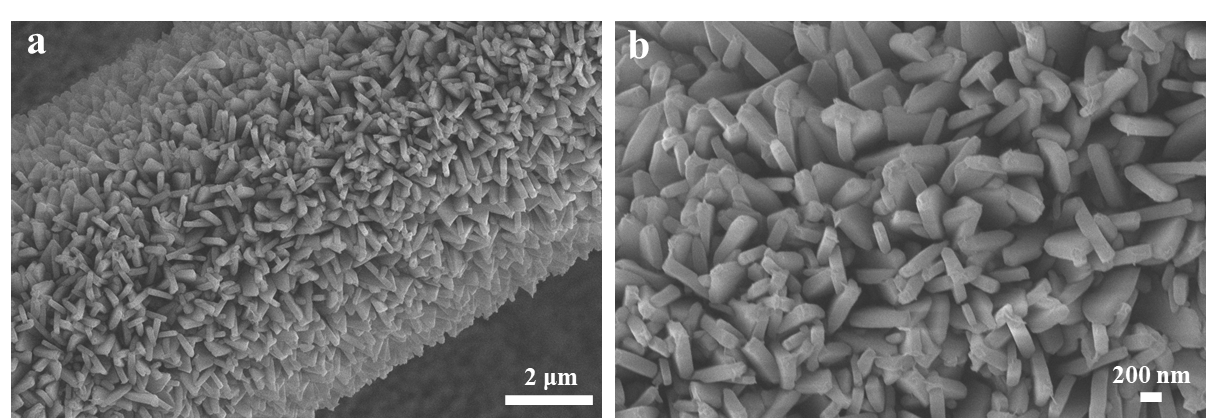


**Figure S2**. The SEM images of Co-MOF/CC.

**
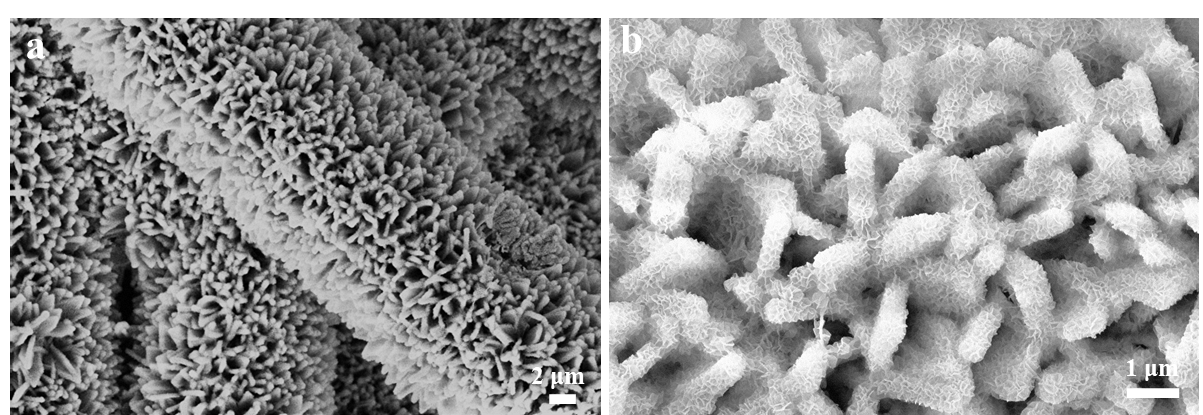
**

**Figure S3**. The SEM images of CoNi-LDH/CC.

**
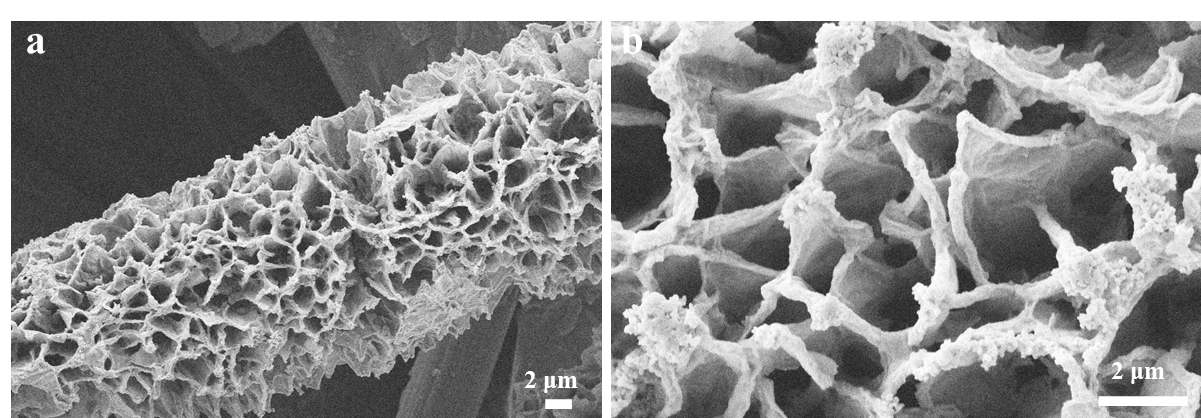
**

**Figure S4**. The SEM images of NC-CoNi_2_S_4_/CC.

**
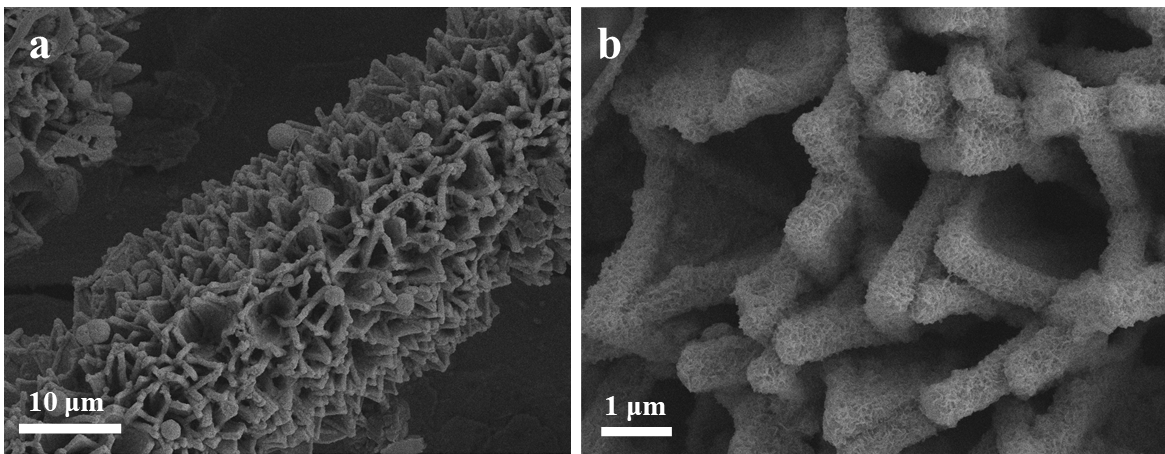
**

**Figure S5**. The SEM images of NC-CoNi_2_S_4_@ReS_2_/CC.

**Figure S6.** N_2_ adsorption-desorption isotherms of NC-CoNi_2_S_4_@ReS_2_/CC.


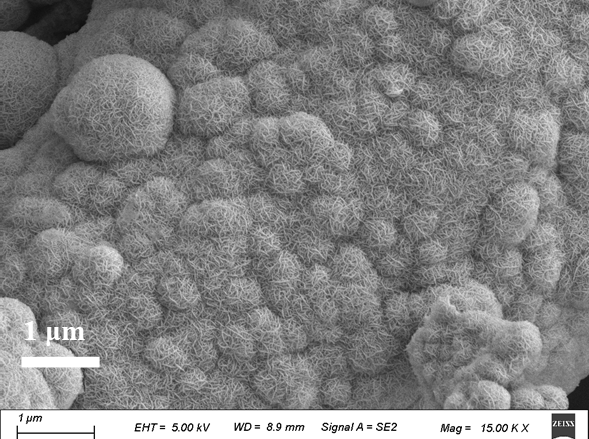


**Figure S7**. The SEM images of ReS_2_/CC.


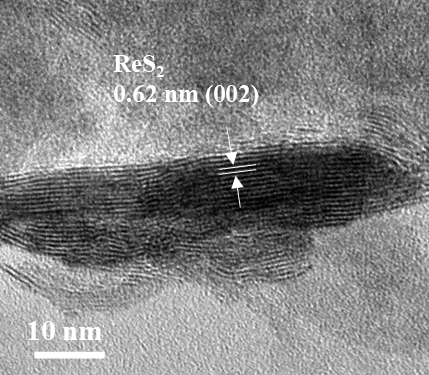


**Figure S8.** HR-TEM of the ReS_2_/CC.

**Figure S9.** EDS of the NC-CoNi_2_S_4_@ReS_2_/CC.

**
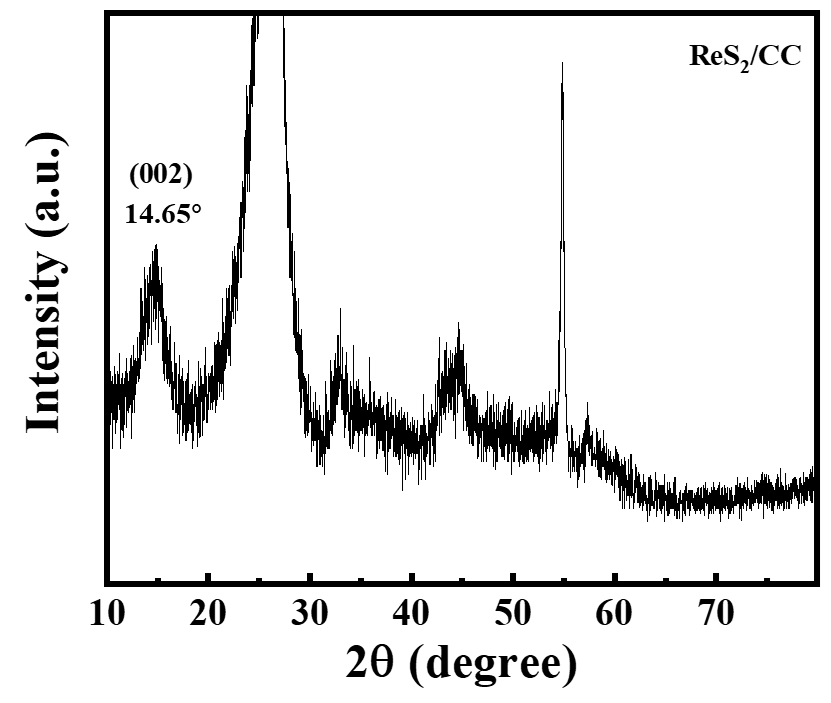
**

**Figure S10.** XRD of the ReS_2_/CC.

**Figure S11.** The full scan XPS spectra of NC-CoNi_2_S_4_@ReS_2_/CC.

**Figure S12.** The high-resolution XPS spectra for C 1s of NC-CoNi_2_S_4_@ReS_2_/CC.

**Figure S13.** The high-resolution XPS spectra for O 1s of NC-CoNi_2_S_4_@ReS_2_/CC.


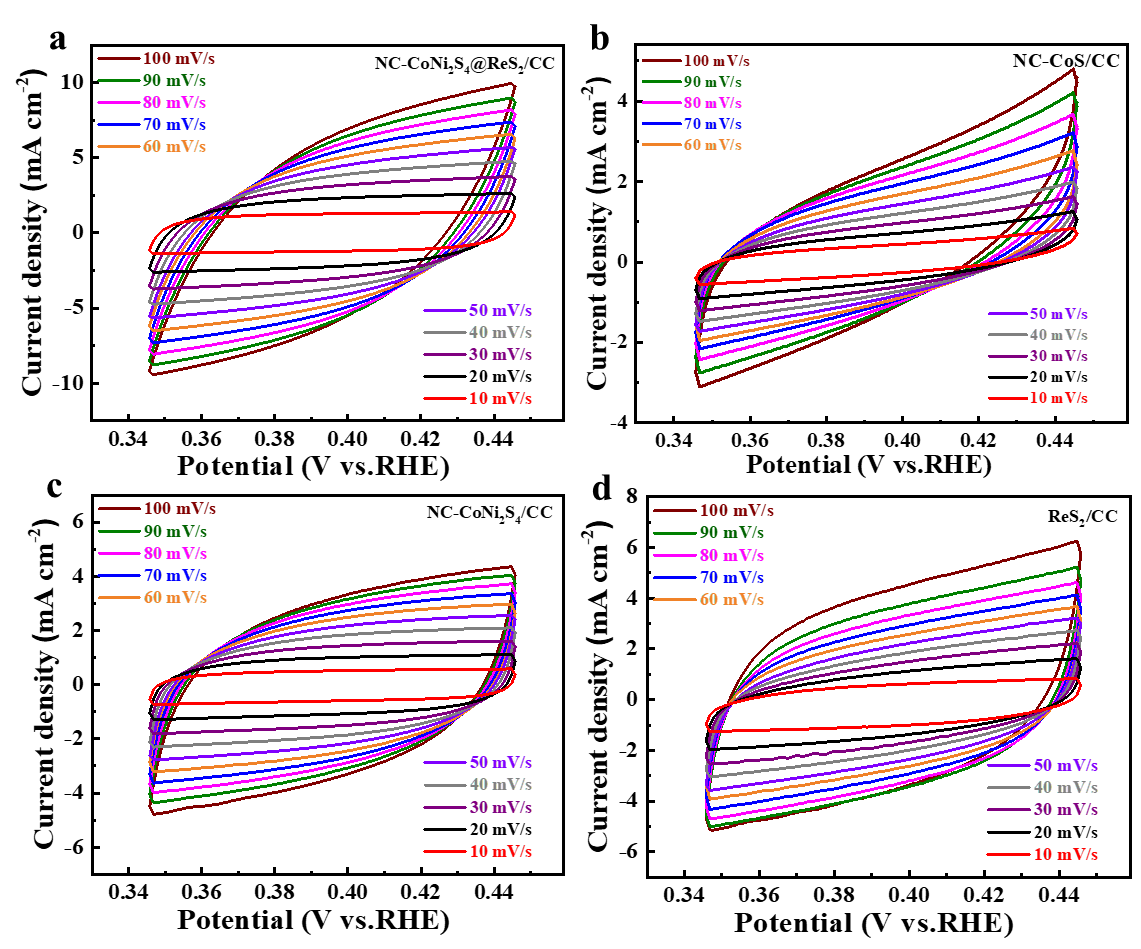


**Figure S14.** Scan-rate dependent CVs of (a) NC-CoNi_2_S_4_@ReS_2_/CC, (b) NC-CoS/CC, (c) NC-CoNi_2_S_4_/CC and (d) ReS_2_/CC in 1M KOH for HER.


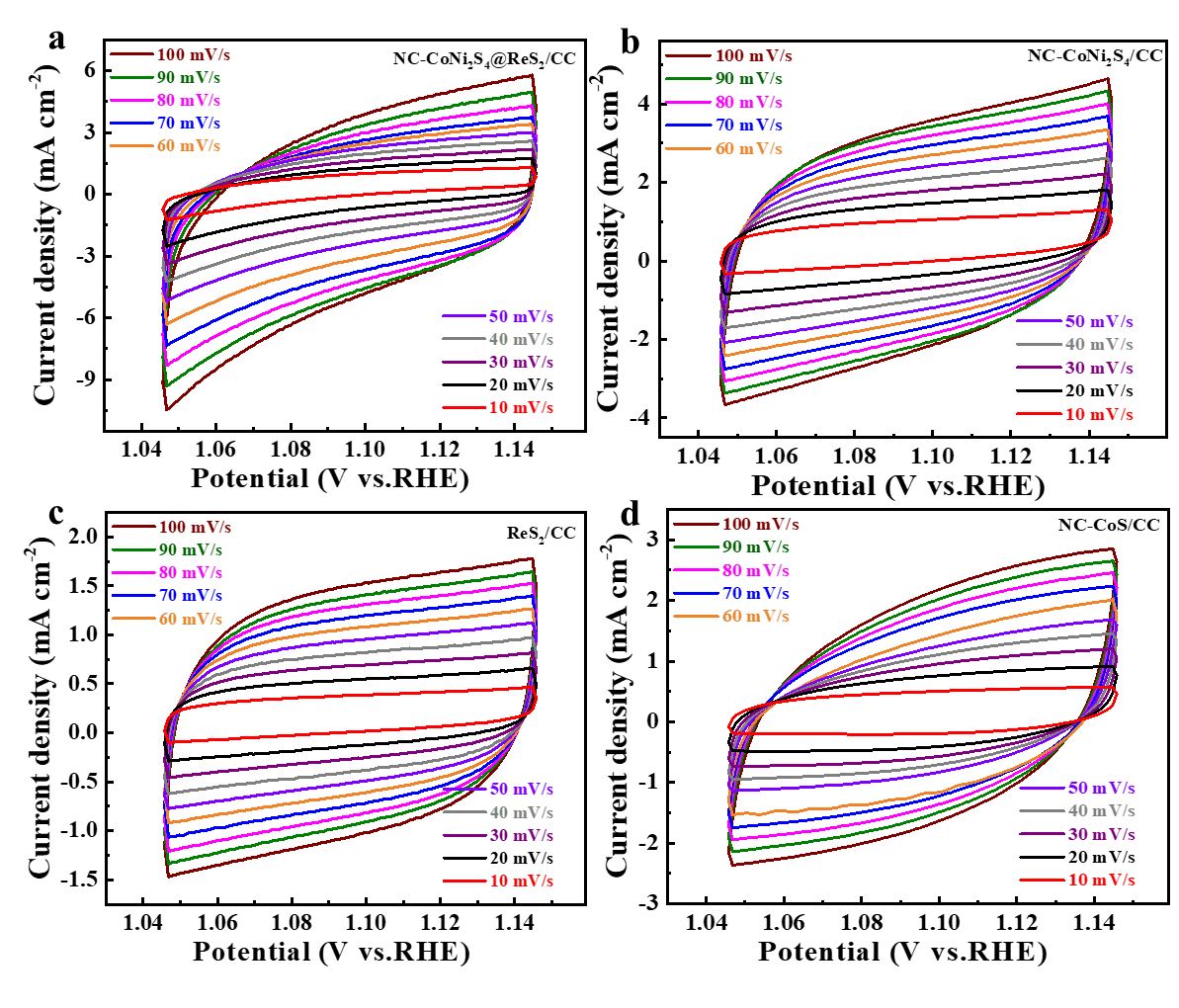


**Figure S15.** Scan-rate dependent CVs of (a) NC-CoNi_2_S_4_@ReS_2_/CC, (b) NC-CoNi_2_S_4_/CC, (c) ReS_2_/CC and (d) NC-CoS/CC in 1M KOH for OER.


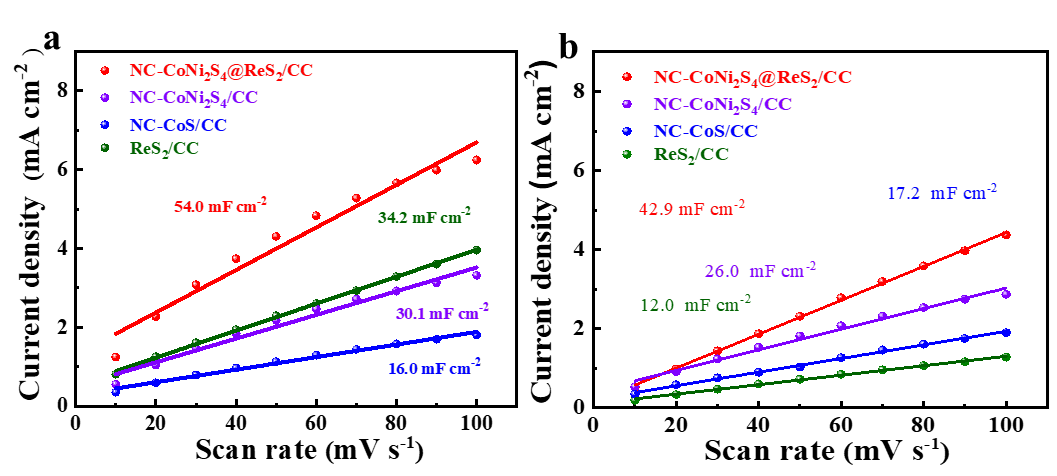


**Figure S16.** The C_dl_ of NC-CoNi_2_S_4_@ReS_2_/CC, NC-CoNi_2_S_4_/CC, NC-CoS/CC and ReS_2_/CC for HER/OER.

**
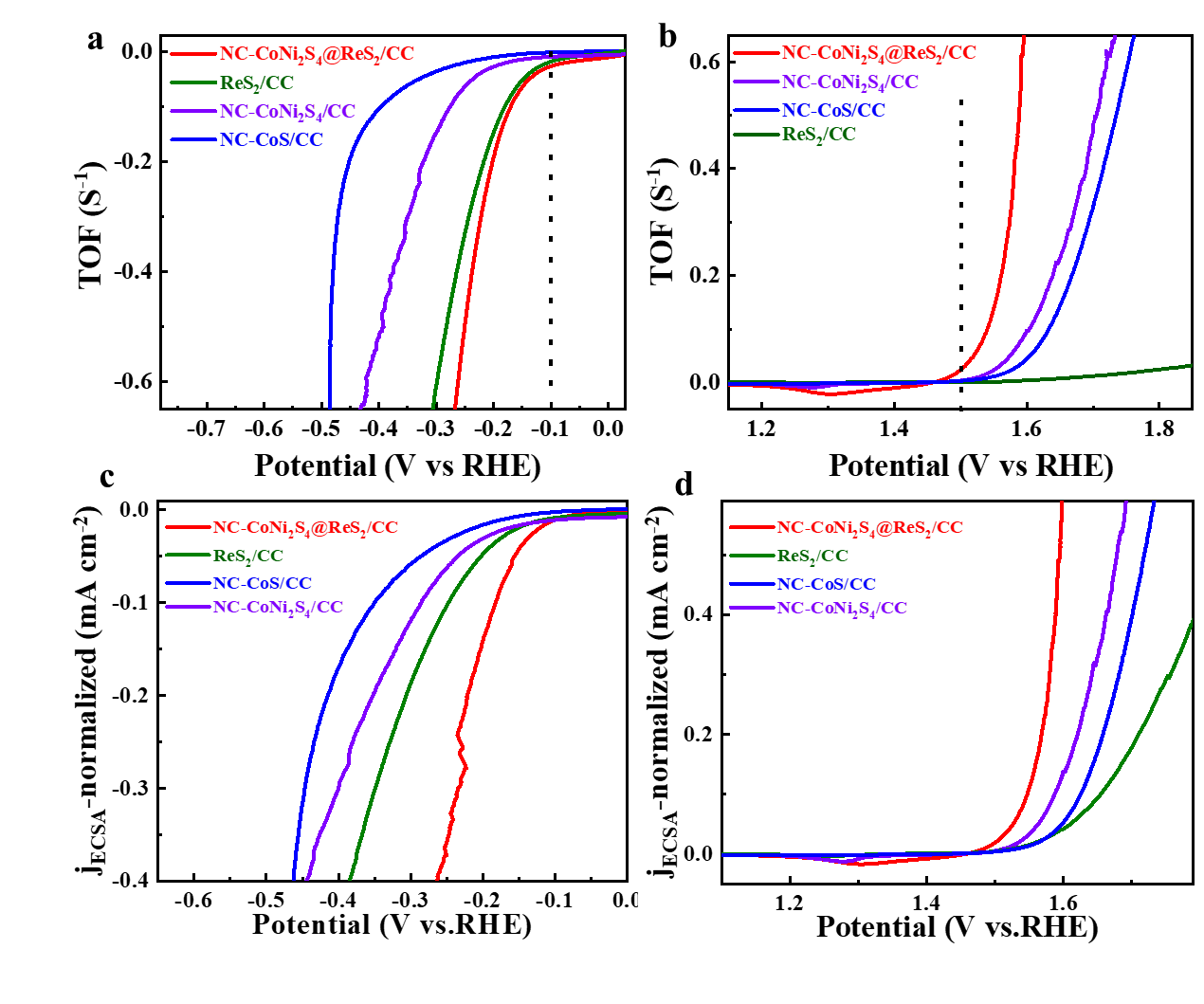
Figure S17.** (a, b) TOF value and (c, d ) LSV normalized by ECSA of NC-CoNi_2_S_4_@ReS_2_/CC, NC-CoNi_2_S_4_/CC, NC-CoS/CC and ReS_2_/CC for HER/OER.

**
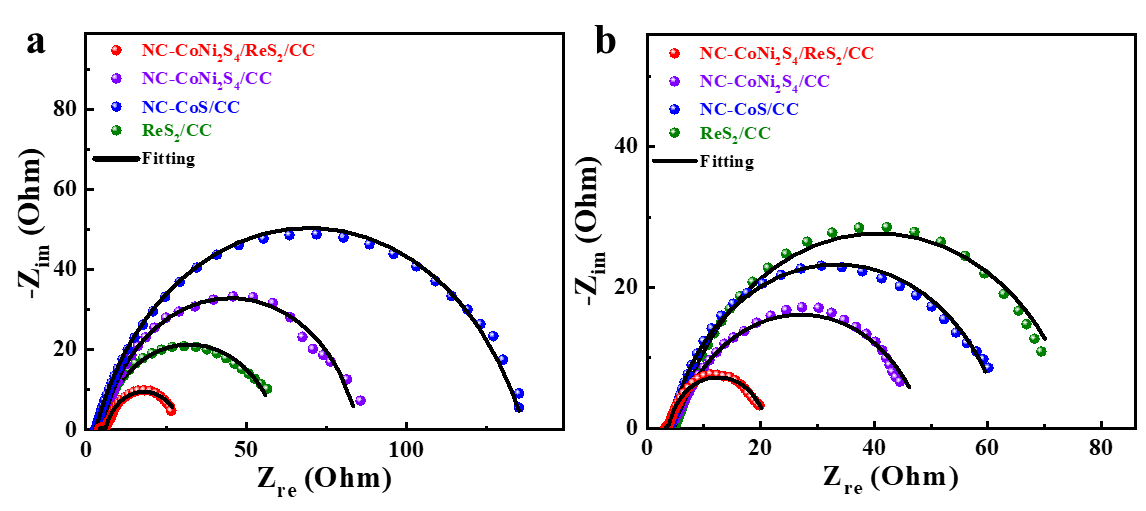
**

**Figure S18.** The EIS spectra of (a-b) NC-CoNi_2_S_4_@ReS_2_/CC, NC-CoNi_2_S_4_/CC, NC-CoS/CC and ReS_2_/CC for HER/OER.


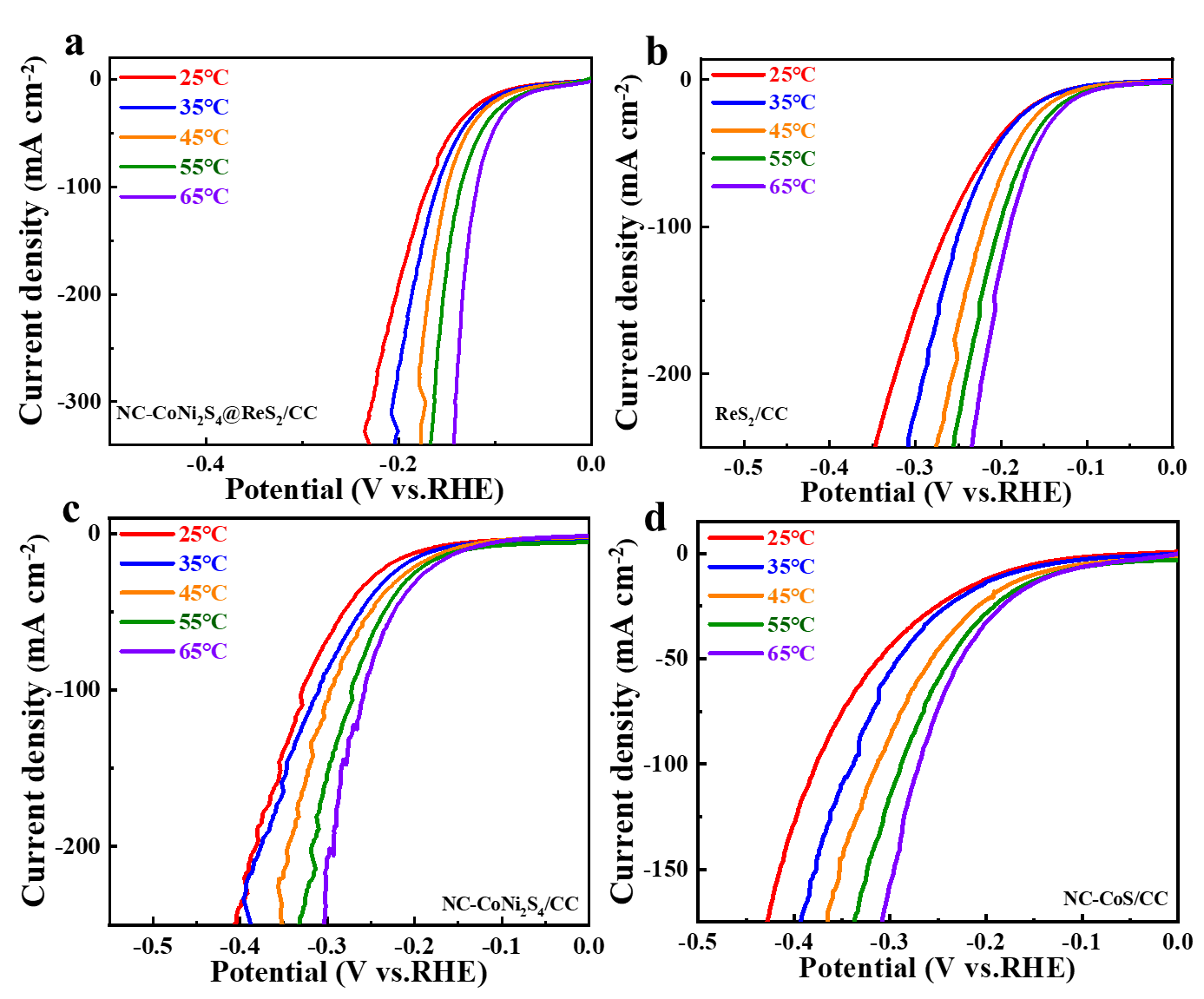


**Figure S19.** LSV curves of NC-CoNi_2_S_4_@ReS_2_/CC, NC-CoNi_2_S_4_/CC, NC-CoS/CC and ReS_2_/CC at various temperatures in 1 M KOH for HER.


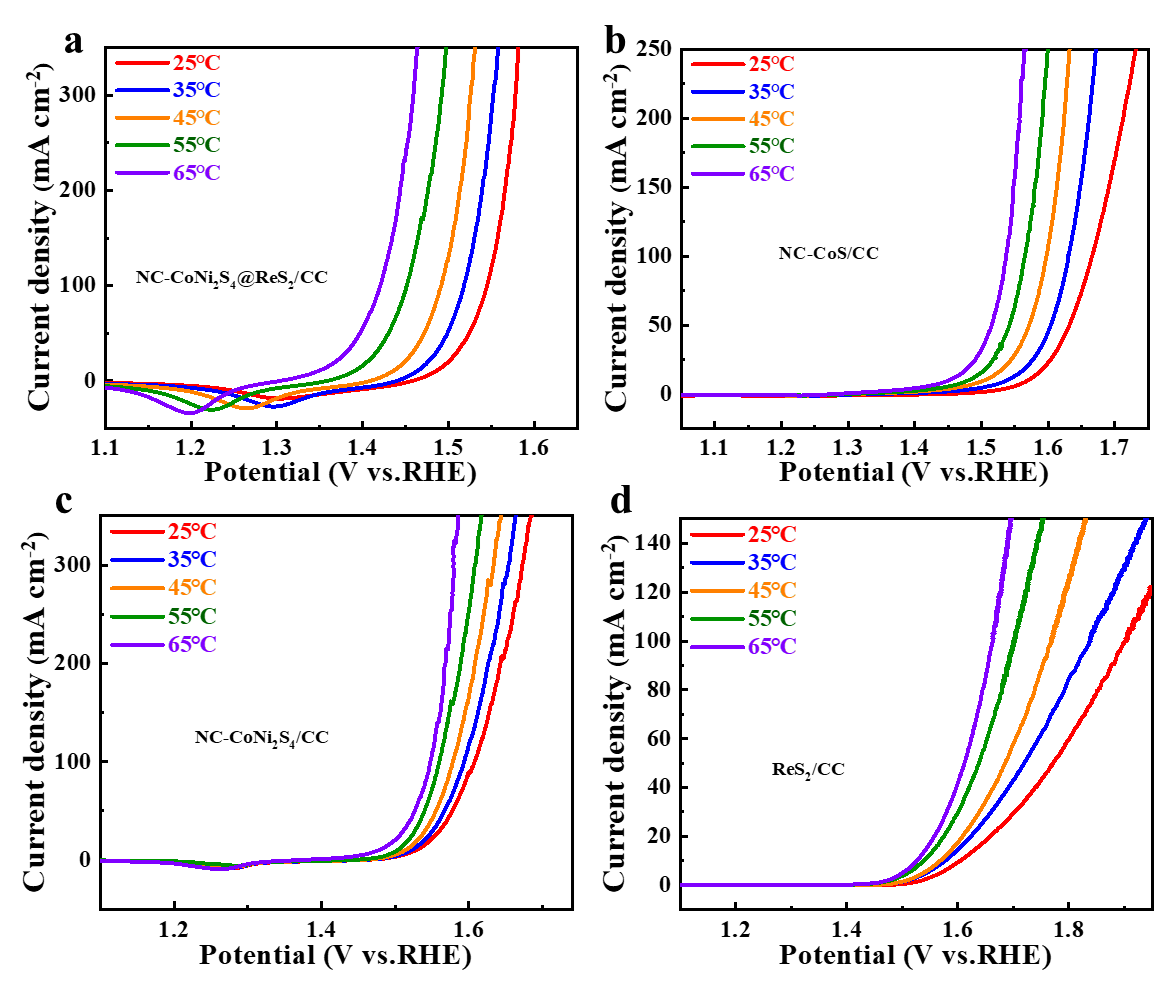


**Figure S20.** LSV curves of NC-CoNi_2_S_4_@ReS_2_/CC, NC-CoNi_2_S_4_/CC, NC-CoS/CC and ReS_2_/CC at various temperatures in 1 M KOH for OER.

**
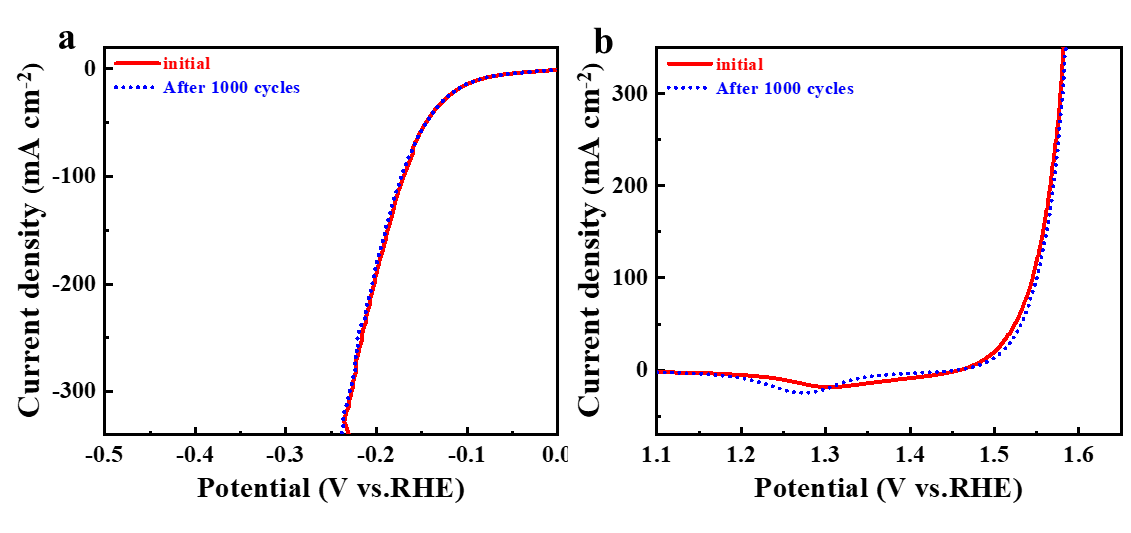
**

**Figure S21.** LSV curves of NC-CoNi_2_S_4_@ReS_2_/CC before and after 1000 CV tests at a scan rate of 5 mV s^-1^.

**
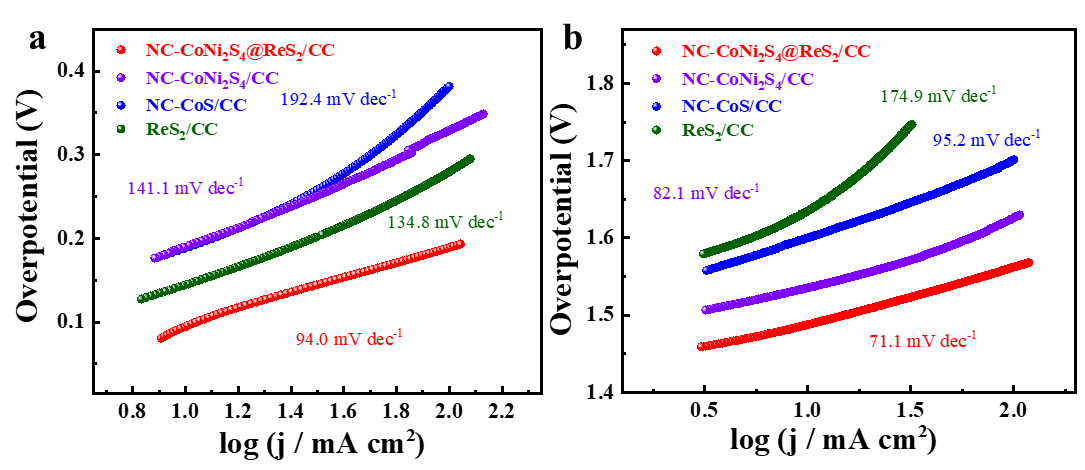
**

**Figure S22.** Tafel plots of NC-CoNi_2_S_4_@ReS_2_/CC, NC-CoNi_2_S_4_/CC, NC-CoS/CC and ReS_2_/CC in 1 M KOH + 0.5 M NaCl for HER/OER.


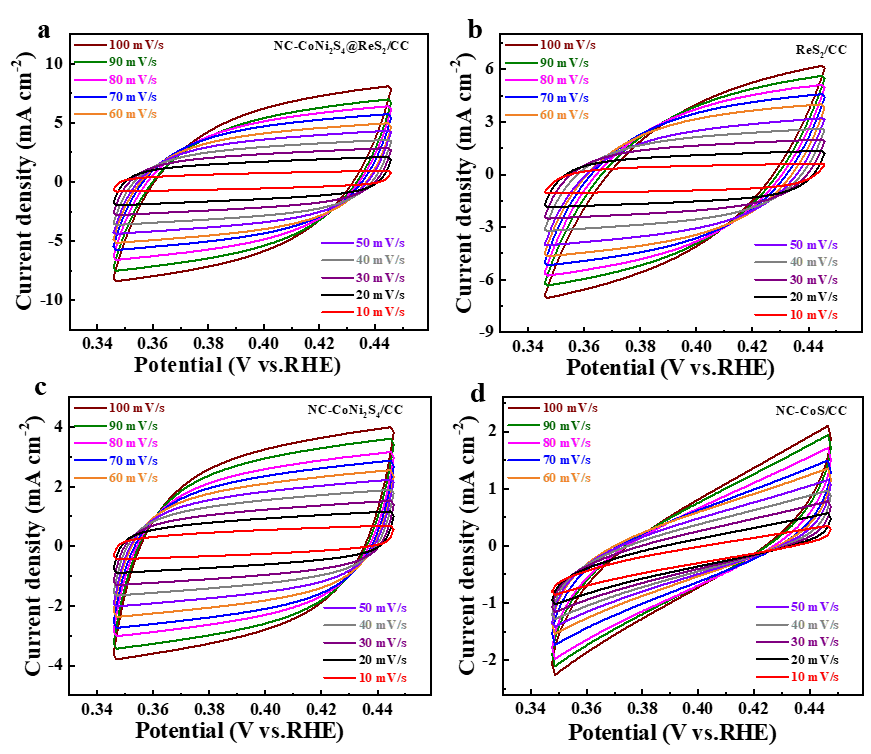


**Figure S23.** Scan-rate dependent CVs of (a) NC-CoNi_2_S_4_@ReS_2_/CC, (b) ReS_2_/CC, (c) NC-CoNi_2_S_4_/CC and (d) NC-CoS/CC in 1M KOH + 0.5 M NaCl for HER.


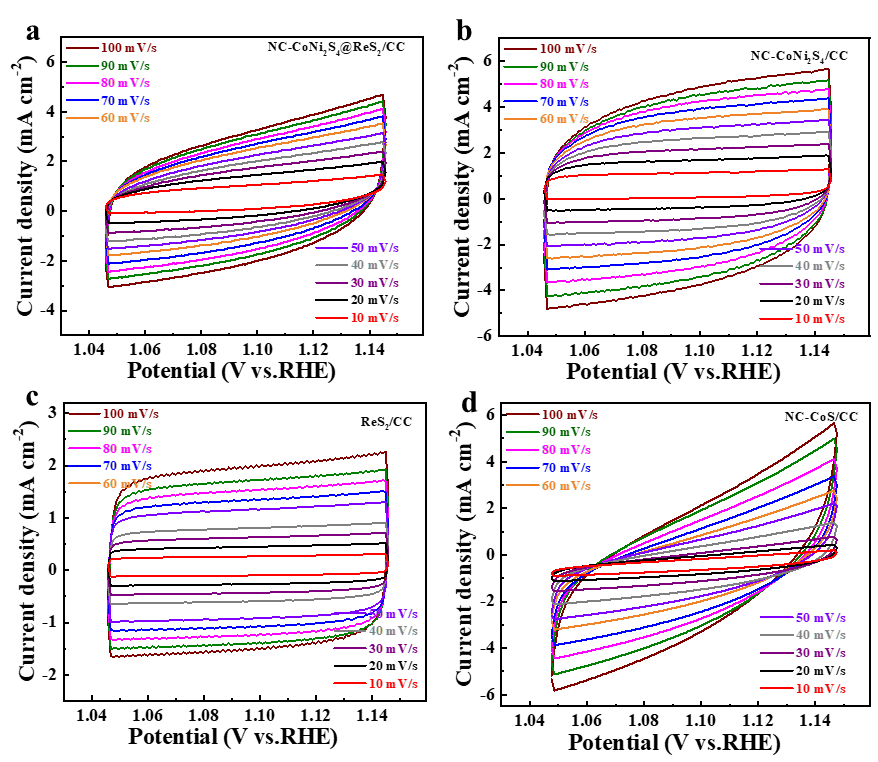


**Figure S24.** Scan-rate dependent CVs of (a) NC-CoNi_2_S_4_@ReS_2_/CC, (b) NC-CoNi_2_S_4_/CC, (c) ReS_2_/CC and (d) NC-CoS/CC in 1M KOH + 0.5 M NaCl for OER.


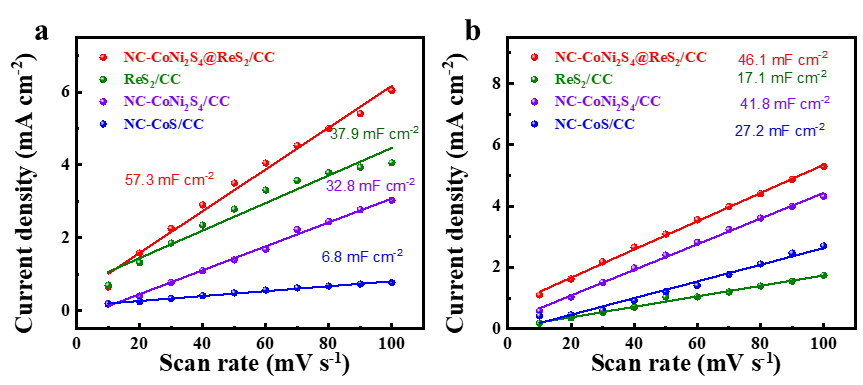


**Figure S25.** The C_dl_ of NC-CoNi_2_S_4_@ReS_2_/CC, NC-CoNi_2_S_4_/CC, NC-CoS/CC and ReS_2_/CC in 1M KOH + 0.5 M NaCl for HER/OER.


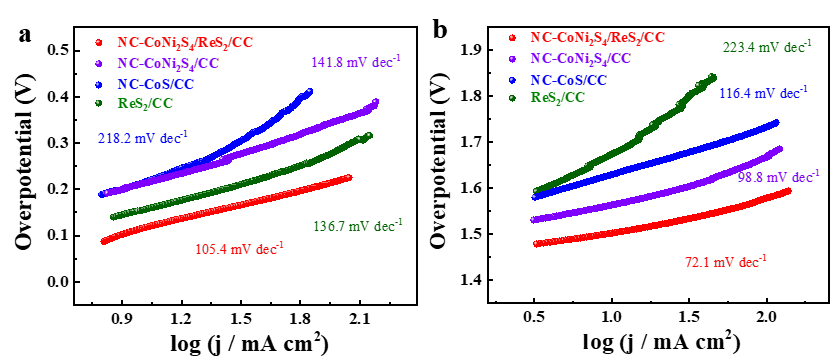


**Figure S26.** Tafel plots of NC-CoNi_2_S_4_@ReS_2_/CC, NC-CoNi_2_S_4_/CC, NC-CoS/CC and ReS_2_/CC in 1 M KOH + seawater for HER/OER.


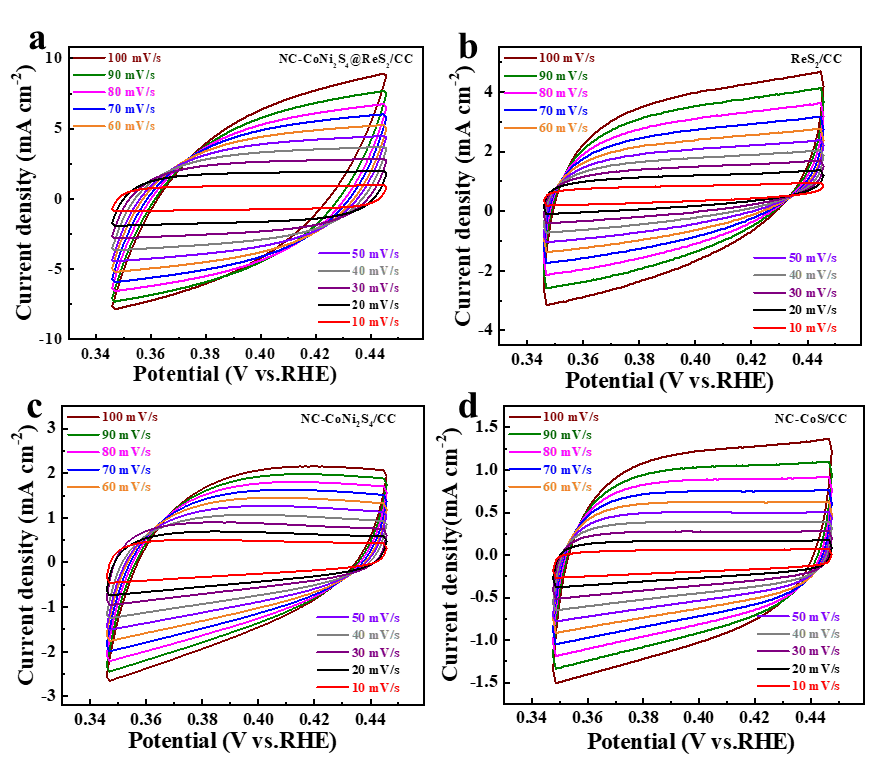


**Figure S27.** Scan-rate dependent CVs of (a) NC-CoNi_2_S_4_@ReS_2_/CC, (b) ReS_2_/CC, (c) NC-CoNi_2_S_4_/CC and (d) NC-CoS/CC in 1M KOH + seawater for HER.


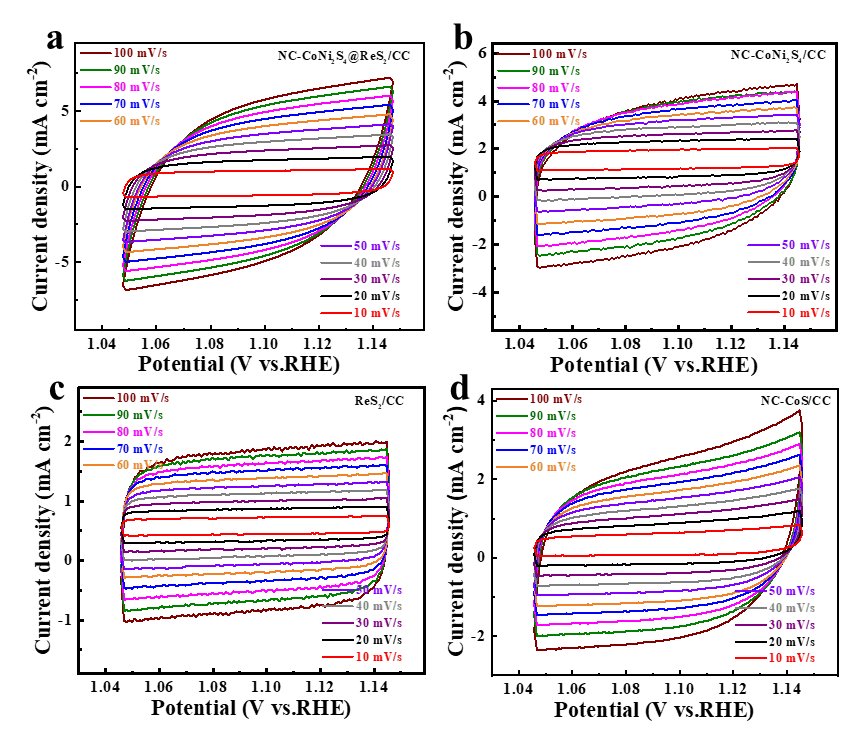


**Figure S28.** Scan-rate dependent CVs of (a) NC-CoNi_2_S_4_@ReS_2_/CC, (b) NC-CoNi_2_S_4_/CC, (c) ReS_2_/CC and (d) NC-CoS/CC in 1M KOH + seawater for OER.


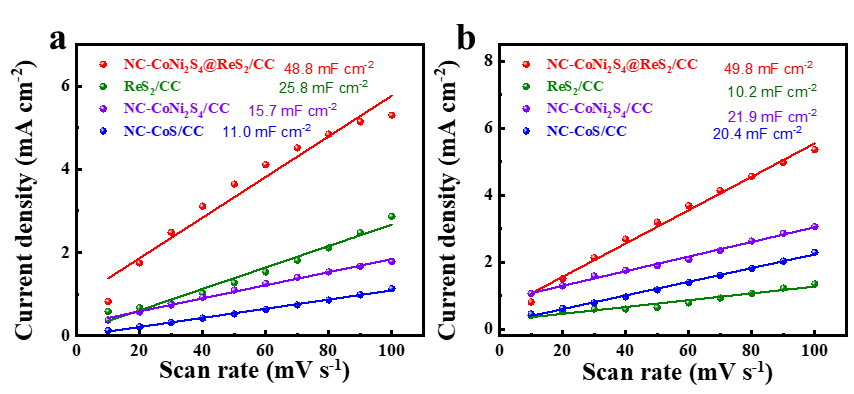


**Figure S29.** The C_dl_ of NC-CoNi_2_S_4_@ReS_2_/CC, NC-CoNi_2_S_4_/CC, NC-CoS/CC and ReS_2_/CC in 1M KOH + seawater for HER/OER.

**
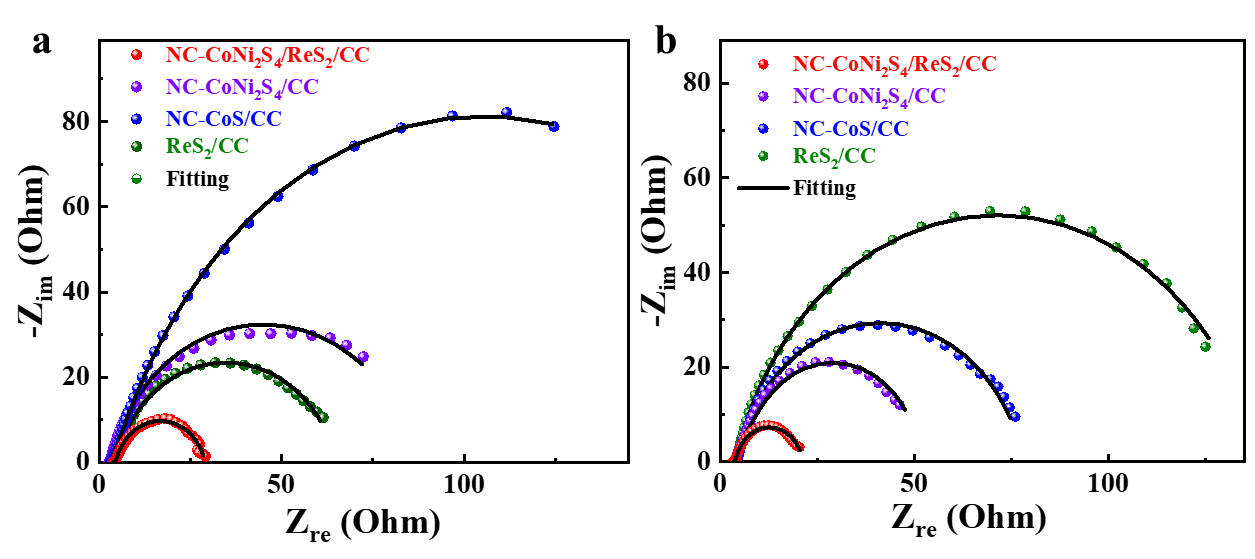
**

**Figure S30.** The EIS spectra of NC-CoNi_2_S_4_@ReS_2_/CC, NC-CoNi_2_S_4_/CC, NC-CoS/CC and ReS_2_/CC in 1 M KOH + 0.5 M NaCl for HER/OER.

**
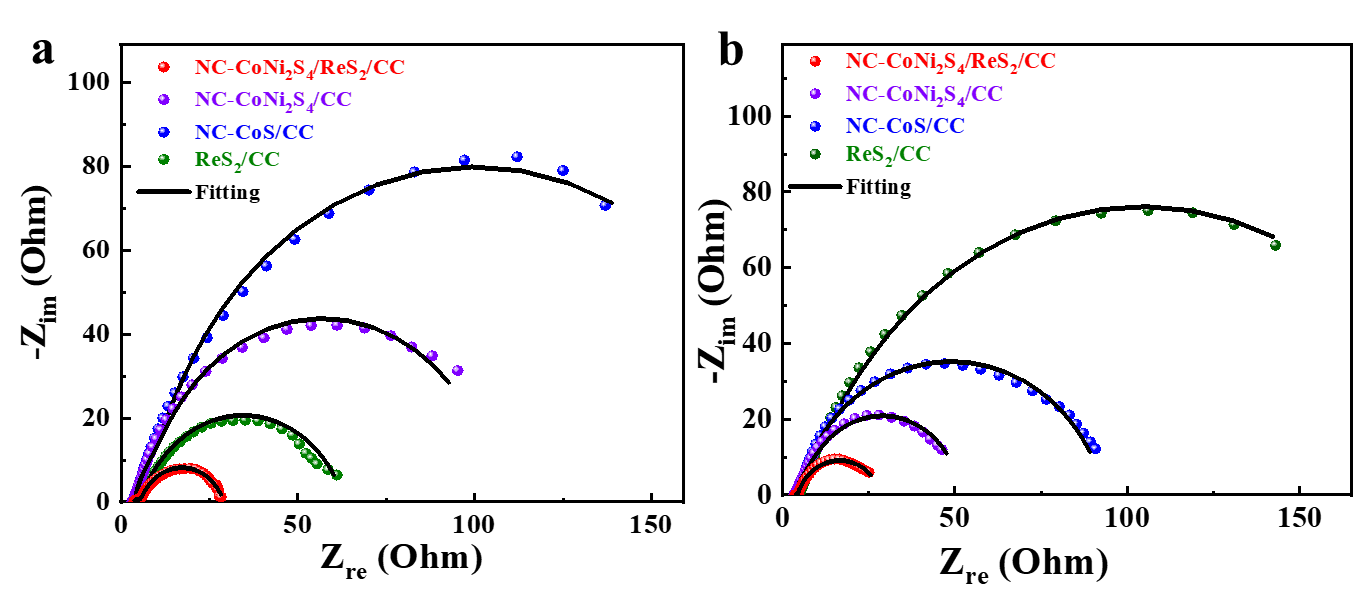
**

**Figure S31.** The EIS spectra of NC-CoNi_2_S_4_@ReS_2_/CC, NC-CoNi_2_S_4_/CC, NC-CoS/CC and ReS_2_/CC in 1 M KOH+seawater for HER/OER.


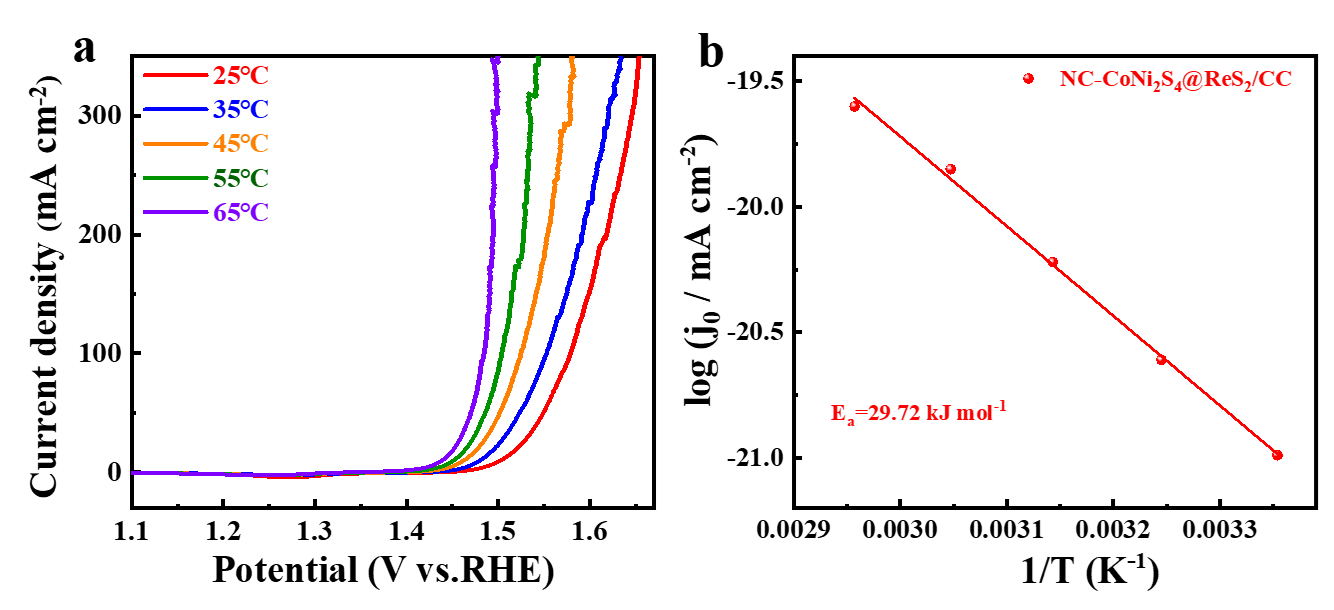


**Figure S32.** (a) LSV curves of NC-CoNi_2_S_4_@ReS_2_/CC at various temperatures in 1 M KOH+ seawater for OER. (b) Arrhenius plots (1/T vs log(j_0_) of NC-CoNi_2_S_4_@ReS_2_/CC.

**
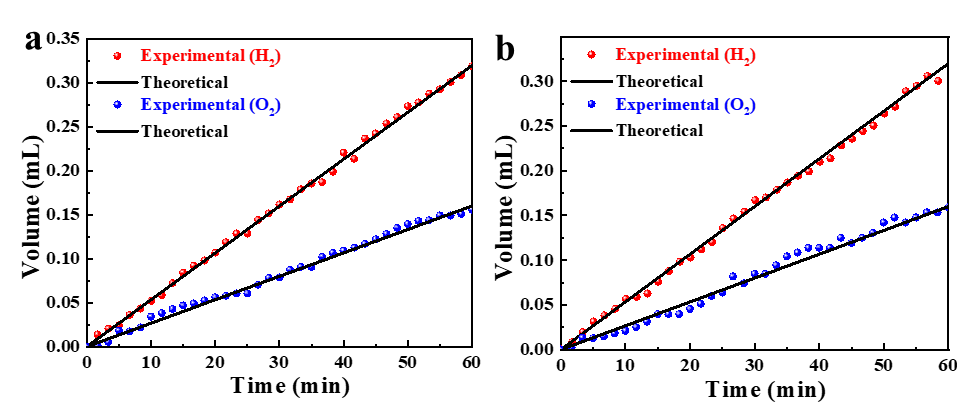
**

**Figure S33.** The Faradic efficiency of NC-CoNi_2_S_4_@ReS_2_/CC in (a) 1 M KOH and (b) 1 M KOH + 0.5 M NaCl.

**Figure S34.** EDS of the NC-CoNi_2_S_4_@ReS_2_/CC after CA test in 1 M KOH+seawater.

**Figure S35.** The full scan XPS of NC-CoNi_2_S_4_@ReS_2_/CC after CA test in 1 M KOH + seawater.

**Figure S36.** XPS of S 2p for NC-CoNi_2_S_4_@ReS_2_/CC after CA test.


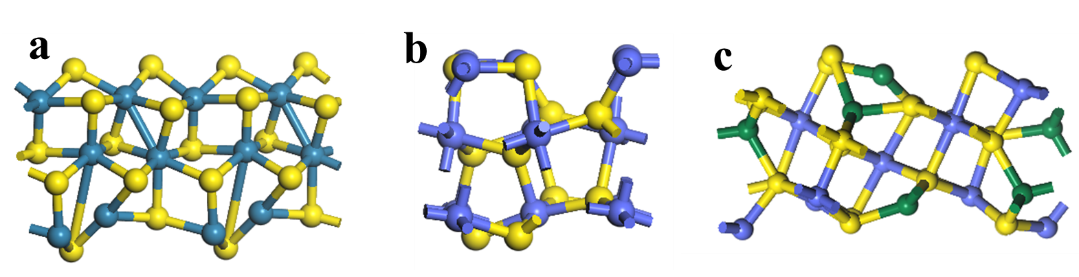


**Figure S37.** Theoretical original model of ReS_2_/CC, NC-CoS/CC and NC-CoNi_2_S_4_/CC.


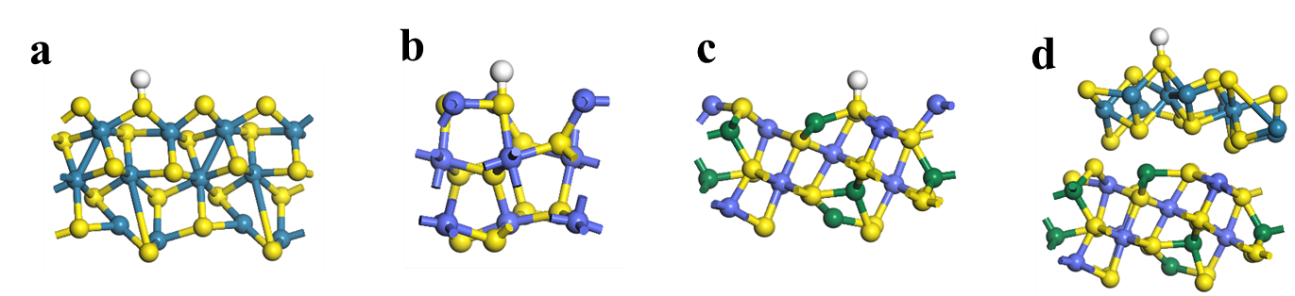


**Figure S38.** Optimized structures models of ReS_2_/CC, NC-CoS/CC NC-CoNi_2_S_4_/CC and NC-CoNi_2_S_4_@ReS_2_/CC for adsorption of *H under HER.


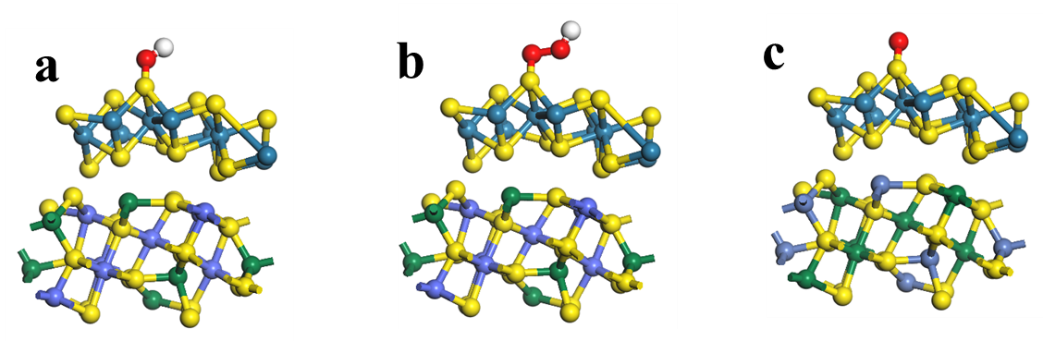


**Figure S39.** Optimized structures models of NC-CoNi_2_S_4_@ReS_2_/CC for adsorption of intermediates (*OH, *O, and *OOH) under OER.


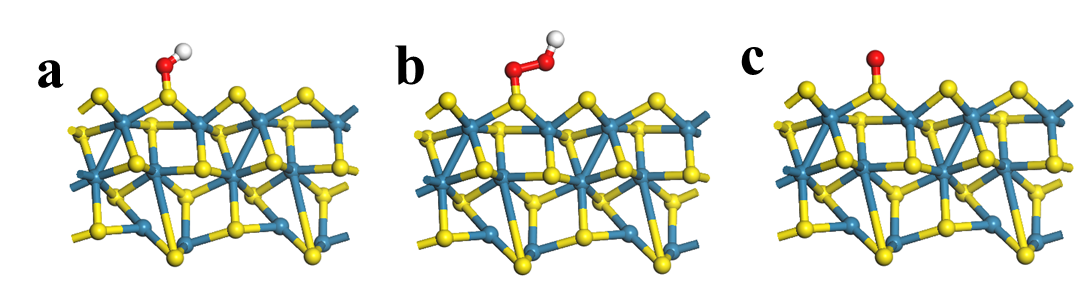


**Figure S40.** Optimized structures models of ReS_2_/CC for adsorption of intermediates (*OH, *O, and *OOH) under OER.


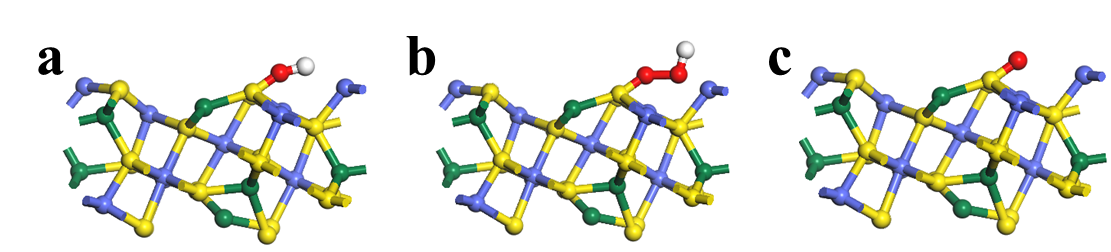


**Figure S41.** Optimized structures models of NC-CoNi_2_S_4_/CC for adsorption of intermediates (*OH, *O, and *OOH) under OER.


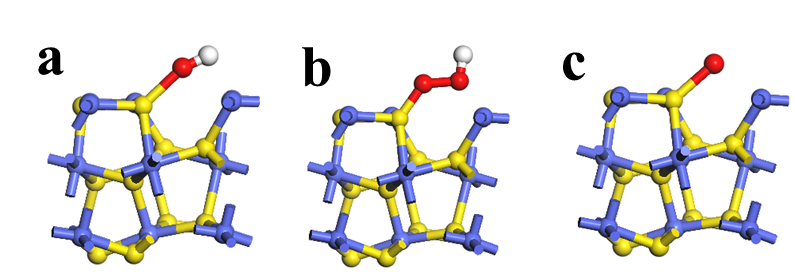


**Figure S42.** Optimized structures models of NC-CoS/CC for adsorption of intermediates (*OH, *O, and *OOH) under OER.

**Table S1.** The values of C_dl_ and ECSA for the HER reaction of different catalysts in 1 M KOH.

| **Catalysts** | **C_dl_ (mF cm^-2^)** | | **ECSA (cm^2^)** |
| --- | --- | --- | --- |
| **NC-CoNi_2_S_4_@ReS_2_/CC** | 54.0 | 1350 | |
| **NC-CoS/CC** | 16.0 | 400 | |
| **NC- CoNi_2_S_4_/CC** | 30.1 | 752.5 | |
| **ReS_2_/CC** | 34.2 | 855 | |

**Table S2.** The values of C_dl_ and ECSA for the OER reaction of different catalysts in 1 M KOH.

| **Catalysts** | **C_dl_ (mF** **cm^-2^)** | | **ECSA (cm^2^)** |
| --- | --- | --- | --- |
| **NC- CoNi_2_S_4_@ReS_2_/CC** | 42.9 | 1072.5 | |
| **NC-CoS/CC** | 17.2 | 430 | |
| **NC- CoNi_2_S_4_/CC** | 26.0 | 650 | |
| **ReS_2_/CC** | 12.0 | 300 | |

**Table S3.** The impedance fitting parameters of the HER reaction of different catalysts in 1 M KOH solution.

| Catalysts | R_s_/Ω | R_ct_/Ω | R_1_/Ω |
| --- | --- | --- | --- |
| NC-CoNi_2_S_4_@ReS_2_/CC | 3.74 | 23.5 | 2.59 |
| NC-CoS/CC | 2.98 | 135.02 | 5.13 |
| NC-CoNi_2_S_4_/CC | 4.14 | 77.22 | 0.44 |
| ReS_2_/CC | 2.96 | 55.55 | 0.32 |

**Table S4.** The impedance fitting parameters of the OER reaction of different catalysts in 1 M KOH solution.

| Catalysts | R_s_/Ω | R_ct_/Ω | R_1_/Ω |
| --- | --- | --- | --- |
| NC-CoNi_2_S_4_@ReS_2_/CC | 3.24 | 17.19 | 0.68 |
| NC-CoS/CC | 3.81 | 58.5 | 0.33 |
| NC-CoNi_2_S_4_/CC | 3.32 | 43.36 | 2.17 |
| ReS_2_/CC | 4.17 | 70.51 | 1.25 |

**Table S5.** the overpotential comparison of the present work with recently reported HER catalysts in 1 M KOH solution.

| Catalysts | Morphology | Overpotential @  10 mA/cm^2^ | Ref |
| --- | --- | --- | --- |
| NC-CoNi_2_S_4_@ReS_2_/CC | Nanosheets | 87 | This work |
| Co@Ni/Fe-MS/MOF | nanorods | 174 | [2] |
| CFMS/NC | Nanosheets | 122 | [3] |
| Co_3_S_4_@NiFe-LDH/NF | Nanosheets | 95 | [4] |
| m-NiTPyP/CNTs | Nanotubes | 138 | [5] |
| NiFeVS_x_@NF | Nanosheets | 127 | [6] |
| MoCoNiS@NF | Nanosheets | 114 | [7] |
| Co_3_O_4_@CoNi_2_S_4_-20/CC | Nnanotube@Nanosheets | 185 | [8] |
| rGO@SN-CoNi_2_S_4_ | Nanosheets | 143 | [9] |
| ReS_2_/CoS | Nanosheets | 187 | [10] |

**Table S6.** The overpotential comparison of the present work with recently reported OER catalysts in 1 M KOH solution.

| Catalysts | Morphology | Overpotential @  10 mA/cm^2^ | Ref |
| --- | --- | --- | --- |
| NC-CoNi_2_S_4_@ReS_2_/CC | Nanosheets | 257 | This work |
| m-NiTPyP/CNTs | Nanotubes | 267 | [5] |
| NiFeVS_x_@NF | Nanosheets | 259 | [6] |
| rGO@SN-CoNi_2_S_4_ | Nanosheets | 310 | [9] |
| NiCo_2_S_4_@S-g-C_3_N_4_ | Nanosheets | 370 | [11] |
| Co_4_Ni_1_S/CC | Nanosheets | 296 | [12] |
| IL-Co@GC-PO | Nanosheets | 340 | [13] |
| FeCoS_y_/NCDs | Nanosheets | 284 | [14] |
| CoFe(OH)F@CoFe_1-x_S-0.05 | Nanosheets | 370 | [15] |
| MXene@CoS/FeS_2_ | Nanosheets | 278 | [16] |

**Table S7.** The impedance fitting parameters of the HER reaction of different catalysts in 1 M KOH + 0.5M NaCl solution.

| Catalysts | R_s_/Ω | R_ct_/Ω | R_1_/Ω |
| --- | --- | --- | --- |
| NC-CoNi_2_S_4_@ReS_2_/CC | 3.68 | 28.42 | 3.71 |
| NC-CoS/CC | 3.10 | 168.8 | 29.18 |
| NC-CoNi_2_S_4_/CC | 2.65 | 77.84 | 0.79 |
| ReS_2_/CC | 3.35 | 60.57 | 1.44 |

**Table S8.** The impedance fitting parameters of the OER reaction of different catalysts in 1 M KOH + 0.5M NaCl solution.

| Catalysts | R_s_/Ω | R_ct_/Ω | R_1_/Ω |
| --- | --- | --- | --- |
| NC-CoNi_2_S_4_@ReS_2_/CC | 3.44 | 17.44 | 0.40 |
| NC-CoS/CC | 3.94 | 73.79 | 0.35 |
| NC-CoNi_2_S_4_/CC | 4.11 | 43.73 | 3.35 |
| ReS_2_/CC | 3.70 | 135.2 | 0.16 |

**Table S9.** The impedance fitting parameters of the HER reaction of different catalysts in 1 M KOH + seawater solution.

| Catalysts | R_s_/Ω | | R_ct_/Ω | R_1_/Ω |
| --- | --- | --- | --- | --- |
| NC-CoNi_2_S_4_@ReS_2_/CC | | 3.72 | 28.17 | 2.66 |
| NC-CoS/CC | 3.98 | | 182.2 | 5.72 |
| NC-CoNi_2_S_4_/CC | 4.04 | | 92.63 | 3.89 |
| ReS_2_/CC | 3.51 | | 60.17 | 1.29 |

**Table S10.** The impedance fitting parameters of the OER reaction of different catalysts in 1 M KOH + seawater solution.

| Catalysts | R_s_/Ω | R_ct_/Ω | R_1_/Ω |
| --- | --- | --- | --- |
| NC-CoNi_2_S_4_@ReS_2_/CC | 3.87 | 24.26 | 0.56 |
| NC-CoS/CC | 4.06 | 89.04 | 0.87 |
| NC-CoNi_2_S_4_/CC | 3.89 | 46.82 | 3.18 |
| ReS_2_/CC | 4.97 | 178.7 | 12.80 |

**Table S11.** comparison of the potentials with the previous reported overall water splitting catalysts.

| Catalysts | Electrolyte | Overpotential  (V) | Ref |
| --- | --- | --- | --- |
| NC-CoNi_2_S_4_@ReS_2_/CC | 1 M KOH | η_10_=1.57, η_100_=1.74 | This work |
|  | 1 M KOH + 0.5 M NaCl | η_10_=1.58, η_100_=1.78 |  |
|  | 1 M KOH + seawater | η_10_=1.6, η_100_=1.83 |  |
| CoFeP(OH)F@CoFe_1-x_S-0.05 | 1 M KOH | η_10_=1.66 | [15] |
|  | 1 M KOH + 0.5 M NaCl | η_10_=1.69 |  |
| NMS | 1 M KOH | η_10_=1.6 | [17] |
| S-FeCo1:1/NF | 1 M KOH | η_10_=1.58, η_50_=1.81 | [18] |
| Ru-CoO_x_/NF\|\|Ru-CoO_x_/NF | 1 M KOH + seawater | η_100_=1.86 | [19] |
| 3%Er-MoO_2_ | 1 M KOH + 0.5 M NaCl | η_10_=1.67 | [20] |
| 1D-Cu@ Co-CoO/Rh | 1 M KOH + 0.5 M NaCl | η_10_=1.6, η_100_=1.85 | [21] |
|  | 1 M KOH + seawater | η_10_=1.7, η_100_=1.95 |  |
| Co_3_S_4_@NiFe-LDH/NF | 1 M KOH | η_10_=1.595 | [4] |
| m-NiTPyP/CNTs | 1 M KOH | η_10_=1.62 | [5] |
| Co-Ni-S/NF | 1 M KOH + 0.5 M NaCl | η_10_=1.67 | [22] |
| Cr-CoxP\|\|Cr-CoxP | 1 M KOH + seawater | η_100_=1.85 | [23] |
| CeO*_x_*@NiCo_2_O_4_/NF | 1 M KOH + 0.5 M NaCl | η_10_=1.66 | [24] |
| Fe_2_O_3_-CoP\|\|P-Fe_2_O_3_-CoP | 1 M KOH + seawater | η_10_=1.65, η_100_=1.82 | [25] |
| Ni_x_B/B_4_C/BCPR/NF | 1 M KOH | η_100_=1.9 | [26] |
| B_5_P_1_\|\|Pt/C/NF | 1 M KOH + seawater | η_100_=1.95 |  |
| NFS | 1 M KOH | η_10_=1.67 | [27] |
|  | 1 M NaOH + 0.5 M NaCl | η_10_=1.84 |  |
| Mo-CoP_X_/NF\|\|Mo-CoP_X_/NF | 1 M KOH + 0.5 M NaCl | η_10_=1.59, η_100_=1.95 | [28] |
|  | 1 M KOH + seawater | η_10_=1.61, η_100_=2.16 |  |
| Zn–NiS-3 | 1 M KOH | η_10_=1.71 | [29] |
| Co_0.33_Fe_0.67_S_2_@NiFe-LDH/CC | 1 M KOH | η_10_=1.58 | [30] |
| NiFeVS_x_@NF | 1 M KOH | η_10_=1.6 | [6] |

**References**

[1] a) G. Kresse, D. Joubert, *Phys. Rev. B* **1999**, *59* (3), 1758; b) J. P. a. B. Perdew, Kieron and Ernzerhof, Matthias, *Phys. Rev. Lett.* **1996**, *77* (18), 3865; c) G. Kresse, J. Furthmüller, *Phys. Rev. B* **1996**, *54* (16), 11169; d) *Comp. Mater. Sci.* **1996**, *6* (1), 15.

[2] S. Yu, N. Zhang, J. Li, J. Yin, Y. Wang, Z. Wu, Y. Zhang, Y. Du, *Acs Sustain. Chem. Eng.* **2024**, *12* (11), 4551.

[3] S. Chen, Y. Zhao, C. Chang, X. Wang, L. Hou, J. Jin, F. Gao, *Fuel* **2024**, *365*, 131230.

[4] L. Chen, H. Chen, L. Wu, G. Li, K. Tao, L. Han, *ACS Appl. Mater. Interf.* **2024**, *16* (7), 8751.

[5] Y. Zhang, S. Chen, Y. Zhang, R. Li, B. Zhao, T. Peng, *Adv. Mater.* **2023**, *35* (19), 2210727.

[6] Y. He, J. Shen, Q. Li, X. Zheng, Z. Wang, L. Cui, J. Xu, J. Liu, *Chem. Eng. J.* **2023**, *474*, 145461.

[7] R. He, P. Thangasamy, J. Wu, K. Yu, X. Yu, W. Tang, D. Quiroz, D. Alyones, Z. Chen, H. Luo, M. Zhou, *Electrochim. Acta* **2023**, *470*, 143342.

[8] Z. Duan, X.-R. Shi, C. Sun, W. Lin, S. Huang, X. Zhang, M. Huang, Z. Yang, S. Xu, *Electrochim. Acta* **2022**, *412*, 140139.

[9] B.-L. Deng, L.-P. Guo, Y. Lu, H.-B. Rong, D.-C. Cheng, *Rare Metals* **2021**, *41* (3), 911.

[10] N. A. Trivedi, P. J. Sharma, K. K. Joshi, V. Patel, C. K. Sumesh, P. M. Pataniya, *Int. J. Hydrogen Energ.* **2024**, *61*, 1212.

[11] H. D. Khalid, A. Bilal, M. Javed, A. Amjad, A. Ali, A. Bahadur, S. Iqbal, S. Mahmood, T. A. Saleh, A. Rana, N. S. Awwad, H. A. Ibrahium, *Int. J. Hydrogen Energ.* **2024**, *68*, 128.

[12] Y. Dong, Z. Fang, W. Yang, B. Tang, Q. Liu, *ACS Appl. Mater. Interf.* **2022**, *14* (8), 10277.

[13] H. Qin, S. Luo, Y. Li, Y. Zhang, R. Dong, Y. Zhou, J. Luo, Y. Zhu, F. Jiang, *Adv. Sustain. Syst.* **2022**, *6* (7), 2200060.

[14] L. Wu, H. Qin, Z. Ji, H. Zhou, X. Shen, G. Zhu, A. Yuan, *Small* **2024**, *20* (4), 2305965.

[15] S. Na, D.-F. Chai, J. Li, S. Chen, X. Yang, S. Fu, G. Sui, D. Guo, *J. Colloid Interf. Sci.* **2024**, *655*, 145.

[16] Z. Zhang, T. Liang, C. Jin, S. Zhang, Y. Cui, J. Chen, X. Zeng, *J. Mater. Chem A.* **2024**, *12* (24), 14517.

[17] S. H. Mujawar, A. S. Salunke, R. J. Deokate, S. T. Salunke, N. K. Shrestha, H. Im, A. I. Inamdar, *Appl. Surf. Sci.* **2024**, *661*, 160081.

[18] Y. Fan, J. Zhang, J. Han, M. Zhang, W. Bao, H. Su, N. Wang, P. Zhang, Z. Luo, *Mater. Horiz.* **2024**, *11* (7), 1797.

[19] D. Wu, D. Chen, J. Zhu, S. Mu, *Small* **2021**, *17* (39), 2102777.

[20] T. Yang, H. Lv, Q. Quan, X. Li, H. Lu, X. Cui, G. Liu, L. Jiang, *Appl. Surf. Sci.* **2023**, *615*, 156360.

[21] P. K. L. Tran, D. T. Tran, D. Malhotra, S. Prabhakaran, D. H. Kim, N. H. Kim, J. H. Lee, *Small* **2021**, *17* (50), 2103826.

[22] S. Gopalakrishnan, V. Saranya, G. Anandha babu, S. Harish, E. Senthil Kumar, M. Navaneethan, *J. Alloys Compd.* **2023**, *965*, 171124.

[23] Y. Song, M. Sun, S. Zhang, X. Zhang, P. Yi, J. Liu, B. Huang, M. Huang, L. Zhang, *Adv. Funct. Mater.* **2023**, *33* (30), 2214081.

[24] W. Liu, J. Zhao, L. Dai, Y. Qi, K. Liang, J. Bao, Y. Ren, *Inorg. Chem.* **2024**, *63* (13), 6016.

[25] Z. Cui, Z. Yan, J. Yin, W. Wang, M.-E. Yue, Z. Li, *J. Colloid Interf. Sci.* **2023**, *652*, 1117.

[26] J. Li, Y. Wang, H. Gao, S. Song, B. Lu, X. Tian, S. Zhou, Y. Yuan, J. Zang, *ChemSusChem* **2021**, *14* (24), 5499.

[27] S. Zhang, Y. Ji, S. Wang, P. Zhang, D. Shi, F. Lu, B. Zhang, *J. Alloys Compd.* **2024**, *1002*, 175323.

[28] Y. Yu, J. Li, J. Luo, Z. Kang, C. Jia, Z. Liu, W. Huang, Q. Chen, P. Deng, Y. Shen, X. Tian, *Mater. Today Nano* **2022**, *18*, 100216.

[29] C. Prakash, P. Sahoo, R. Yadav, A. Pandey, V. K. Singh, A. Dixit, *Int. J. Hydrogen Energ.* **2023**, *48* (58), 21969.

[30] G. Huang, X. Gao, Y. Liu, S. Yuan, Y. Zhang, N. Li, S. Jing, *Electrochim. Acta* **2023**, *449*, 142219.
